# A Bioinspired Nonheme Fe^III^–(O_2_^2–^)–Cu^II^ Complex with an *S*_t_ = 1 Ground State

**DOI:** 10.1021/jacs.4c04492

**Published:** 2024-07-05

**Authors:** Dustin Kass, Sagie Katz, Hivda Özgen, Stefan Mebs, Michael Haumann, Ricardo García-Serres, Holger Dau, Peter Hildebrandt, Thomas Lohmiller, Kallol Ray

**Affiliations:** † Institut für Chemie, Humboldt-Universität zu Berlin, Brook-Taylor-Straße 2, 12489 Berlin, Germany; ‡ Department of Chemistry, Technische Universität Berlin, Straße des 17. Juni 135, 10623 Berlin, Germany; § Department of Physics, Freie Universität Berlin, Arnimallee 14, 14195 Berlin, Germany; ∥ Université Grenoble Alpes, CEA, CNRS, Laboratoire de Chimie et Biologie des Métaux, 38000 Grenoble, France; ⊥ EPR4Energy Joint Lab, Department Spins in Energy Conversion and Quantum Information Science, Helmholtz-Zentrum Berlin für Materialien und Energie GmbH, Albert-Einstein-Straße 16, 12489 Berlin, Germany

## Abstract

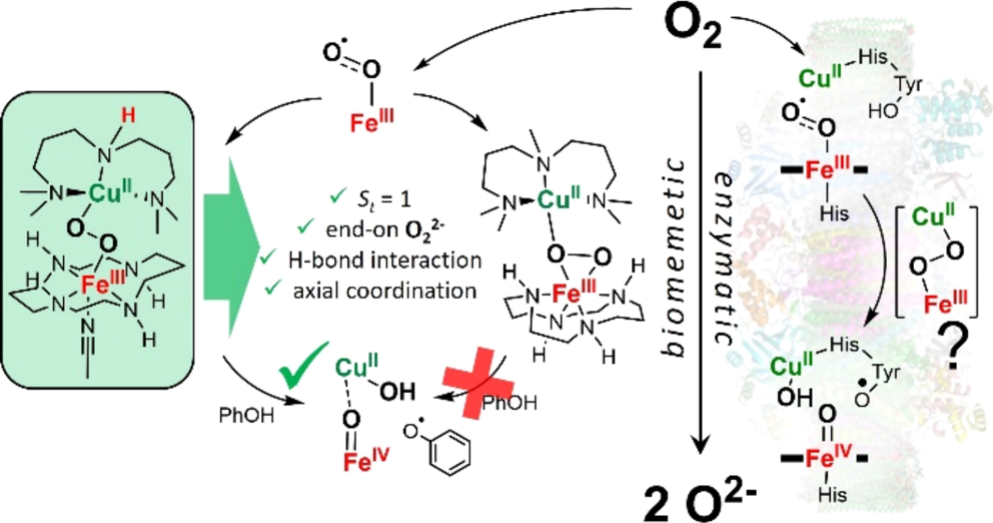

Cytochrome *c* oxidase (CcO) is a heme copper oxidase (HCO) that catalyzes the natural reduction of oxygen to water. A profound understanding of some of the elementary steps leading to the intricate 4e^–^/4H^+^ reduction of O_2_ is presently lacking. A total spin *S*_t_ = 1 Fe^III^–(O_2_^2–^)–Cu^II^ (**I_P_**) intermediate is proposed to reduce the overpotentials associated with the reductive O–O bond rupture by allowing electron transfer from a tyrosine moiety without the necessity of any spin-surface crossing. Direct evidence of the involvement of **I**_**P**_ in the CcO catalytic cycle is, however, missing. A number of heme copper peroxido complexes have been prepared as synthetic models of **I**_**P**_, but all of them possess the catalytically nonrelevant *S*_t_ = 0 ground state resulting from antiferromagnetic coupling between the *S* = 1/2 Fe^III^ and Cu^II^ centers. In a complete nonheme approach, we now report the spectroscopic characterization and reactivity of the Fe^III^–(O_2_^2–^)–Cu^II^ intermediates **1** and **2**, which differ only by a single −CH_3_ versus −H substituent on the central amine of the tridentate ligands binding to copper. Complex **1** with an end-on peroxido core and ferromagnetically (*S*_t_ = 1) coupled Fe^III^ and Cu^II^ centers performs H-bonding-mediated O–O bond cleavage in the presence of phenol to generate oxoiron(IV) and exchange-coupled copper(II) and PhO^•^ moieties. In contrast, the μ-η^2^:η^1^ peroxido complex **2**, with a *S*_t_ = 0 ground state, is unreactive toward phenol. Thus, the implications for spin topology contributions to O–O bond cleavage, as proposed for the heme Fe^III^–(O_2_^2–^)–Cu^II^ intermediate in CcO, can be extended to nonheme chemistry.

## Introduction

**Scheme 1:**
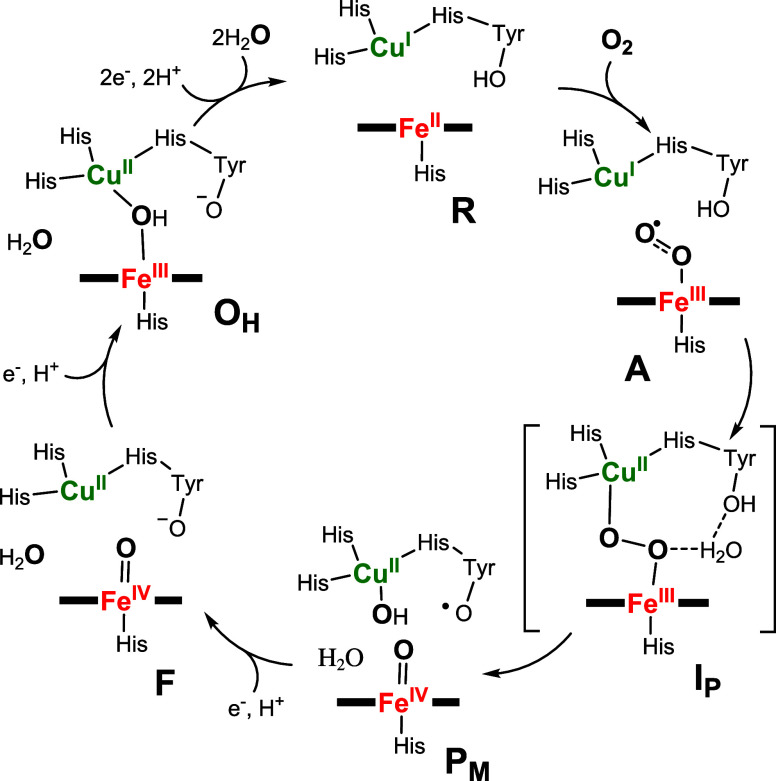
Schematic Cycle of the Oxygen Reduction by CcO and the Involved Intermediate States

Cytochrome *c* oxidase (CcO) is a multisubunit transmembrane protein, which catalyzes the 4e^–^/4H^+^ reduction of dioxygen to water during the last step of the electron-transport chain, thereby generating a transmembrane proton gradient responsible for driving ATP synthesis.^1−3^ CcO contains a unique heterobimetallic heme copper active site, the metal centers of which are separated by >4.0 Å.^4,5^ The four electrons required to fully reduce dioxygen to water are supplied by iron (Fe^II^ → Fe^IV^), copper (Cu^I^ → Cu^II^), and a tyrosine residue (Tyr–OH → Tyr–O^•^), which is covalently tethered to one of the histidine moieties ligated to Cu. The consensus mechanism of CcO, shown in Scheme 1, involves the initial binding of dioxygen to the reduced (**R**) form of the enzyme to form transient intermediate **A**, which is then rapidly converted to **P**_**M**_.^3,5^ The electronic structure of **P**_**M**_ is unambiguously assigned as a ferryl-heme-cupric-hydroxide-tyrosyl-radical species based on various spectral and chemical evidence.^6^ However, the identity of the species formed before the O–O cleaved intermediate **P**_**M**_ has proved controversial. For example, intermediate **A**, which is believed to formally be a ferric-superoxido species, exhibits a ν(Fe–O) stretch of 571 cm^–1^,^7^ which overlaps with end-on (hydro)peroxidos found in proteins^8−10^ and various well-characterized synthetic low-spin heme copper peroxido complexes in organic media or on electrode surfaces.^5,11−15^ Accordingly, the lack of empirical vibrational data does not allow for a definite assessment of the degree of O_2_ reduction for intermediate **A**. Furthermore, various theoretical and kinetic studies have hypothesized the involvement of a total spin *S*_t_ = 1 heme Fe^III^–O_2_–Cu^II^ peroxido intermediate (**I**_**P**_) during the **A** to **P**_**M**_ conversion;^16−19^ however, direct spectroscopic evidence of such intermediate in the CcO catalytic cycle has stayed elusive. Notably, the ferromagnetic coupling between the low-spin *S* = 1/2 Fe^III^ and *S* = 1/2 Cu^II^ centers in the *S*_t_ = 1 heme Fe^III^–O_2_–Cu^II^ peroxido intermediate to form the exchange-coupled Cu^II^–OH and Tyr^•^ products in CcO is discussed as the prerequisite necessary for the efficient Tyr-mediated reductive O–O bond rupture without any spin-surface crossing.^18,20,28^

Synthetic examples that demonstrate the structural feasibility of the proposed heme Fe^III^–O_2_–Cu^II^ peroxido intermediate in CcO have been reported.^5,11,12,21−28^ Nevertheless, the acute Fe–O–O–Cu dihedral angle in all of these complexes have led to a catalytically nonrelevant antiferromagnetically coupled *S*_t_ = 0 ground state of the low-spin heme Fe^III^–O_2_–Cu^II^ adducts.^14,15^ The presence of a bridging heme-peroxido-copper species has also been reported in the X-ray structures of the resting state of CcO.^4,29−31^ But the exact nature of the O_2_-reduced moiety and its formation in these “as isolated” structures have been disputed. In particular, the reported O–O and Fe/Cu–O bond lengths in these structures are inconsistent with the peroxido assignment.^5^ One of the “as isolated” structures was reported to exhibit a UV–vis absorption feature at 650 nm, which when excited at this wavelength showed a resonance Raman (rRaman) active band at 755 cm^–1^.^32^ This band, which was initially thought to be a ν(O–O) stretching mode, was later assigned to a mode of the His-419 ligand based on rRaman spectroscopy.^33^ This provided further doubt to the proposed binding of the peroxido ligand in the “as isolated” form of CcO.

Herein, we report the synthesis, spectroscopic characterization, and reactivity of a nonheme [(CH_3_CN)(*trans*-cyclam)Fe^III^(μ-η^1^*:*η^1^*-*O_2_)Cu^II^(AN)]^3+^ (**1**; AN = 3,3′-iminobis(*N*,*N*-dimethylpropyl-amine)) complex, which may improve our understanding of the properties of the elusive catalytically relevant ferromagnetically coupled (*S*_t_ = 1) low-spin heme Fe^III^–O_2_–Cu^II^ peroxido intermediate proposed during the **A** to **P**_**M**_ conversion in CcO. Notably, the corresponding complex [(*cis*-cyclam)Fe^III^(μ-η^2^*:*η^1^-O_2_)Cu^II^(MeAN)]^3+^ (**2**; MeAN = 2,6,10-trimethyl-2,6,10-triazaundecane), where MeAN differs from AN in having one methyl group in the ligand structure, exhibits distinct O_2_ binding mode and reactivity properties different from **1**. The present study, therefore, highlights the importance of the interplay between metal centers and the local environment in governing key physical and chemical properties of biologically relevant dinuclear metal-dioxygen intermediates.

## Results and Discussion

### Synthesis and UV–Vis and Mössbauer Characterizations of **1**

**Figure 1:**
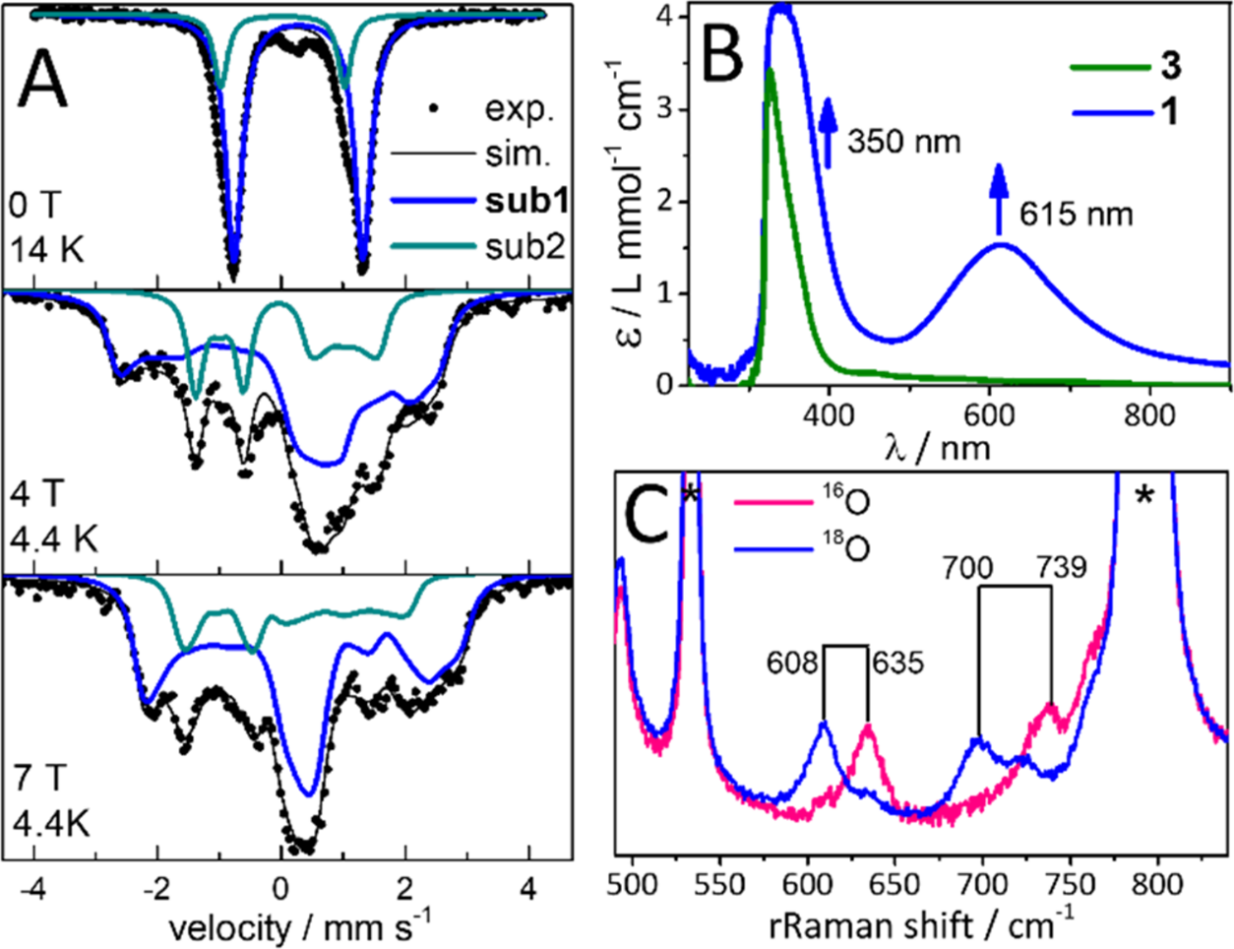
(A) Mößbauer spectra at variable magnetic field (parallel to the γ-beam) of ^57^Fe-enriched **1** in a frozen solution of an acetone/CH_3_CN 10:1 v/v mixture. The simulation (black lines) of the experimental data (dots) gave a best fit with two subspecies, sub1 (blue lines) with *S*_t_ = 1 corresponding to **1** (70%, δ = 0.26 mm s^–1^, Δ*E*_Q_ = −2.11 mm s^–1^, *D*_t_ = 1.5 cm^–1^, *A*_*xx*_ = −3.2 T, *A*_*yy*_ = −16.2 T, *A*_*zz*_ = 4.4 T, η = 0.43) and sub2 (green lines) corresponding to a partially formed (24%) Fe^IV^ (*S* = 1) species (see Figure S1 for further details); please note that the high-spin Fe^III^ signal observed in electron paramagnetic resonance (EPR) (Figure S3) is not detected in Mössbauer; (B) UV–vis absorption spectra of **1** (blue) and **3** (green) in a 10:1 acetone/CH_3_CN v/v mixture at −90 °C. (C) rRaman spectra of **1** (pink) and ^18^O-labeled **1** (blue) measured in a 10:1 acetone/CH_3_CN v/v solution (407 nm excitation, 2 mW, −90 °C). Solvent features are marked by asterisks.

In a previous study, we reported the formation and characterization of the [(CH_3_CN)(*trans*-cyclam)Fe^III^(O_2_^–•^)]^2+^ compound **3** by addition of O_2_ to a solution of [(CH_3_CN)_2_(*trans*-cyclam)Fe^II^](OTf)_2_.^34^Figure 1B shows the UV–vis spectrum after reaction of one equivalent of the well-characterized [Cu^I^(AN)]BF_4_ complex^28,35^ with **3**, which led to the formation of a deep blue species, **1**, with an absorption maximum at 615 nm (ε = 1.5 L mmol^–1^ cm^–1^) and a half-life time *t*_1/2_ = 10 min at −90 °C. The 14 K Mößbauer spectrum of **1** (Figure 1A) shows a main species in ∼70% yield with parameters (isomer shift δ = 0.26 mm s^–1^, quadrupole splitting |Δ*E*_Q_| = 2.11 mm s^–1^) that are typical for a low-spin (*S* = 1/2) Fe^III^ center. However, the low-temperature, low-field (5 K, 0.06 T) Mößbauer spectrum of **1** (Figure S1, top, blue lines) does not display a magnetic splitting but instead a broadened, asymmetric doublet, characteristic of an integer spin with a positive zero-field splitting (ZFS) parameter. This is confirmed by applied magnetic field Mößbauer studies of **1** (Figure 1A), where the spectra are best fit in the slow-relaxation limit with a total spin *S*_t_ = 1 with a small axial zero-field splitting *D*_t_ = 1.5 cm^–1^ and an anisotropic hyperfine coupling tensor. The asymmetry parameter η = 0.43 determined for **1** differs from the reported parameters for the axially symmetric [Fe^IV^(O)(*trans*-cyclam)(CH_3_CN)]OTf_2_ (η = 0) complex.^34^ The Mössbauer spectra of the minor species (Figure S1, green lines) are completely compatible with an axial Fe^IV^=O complex. Altogether, the Mößbauer measurements propose a species with a low-spin Fe^III^ that is ferromagnetically coupled to a Cu^II^ center, giving rise to a *S*_t_ = 1 state in **1**.

### X-ray Absorption Near-Edge Structure (XANES) and EPR Spectroscopy

In addition to Fe^III^, the presence of a Cu^II^ center **1** is confirmed by the X-ray absorption near-edge structure (XANES) data (Figure S2). The XANES spectra at the Cu K-edge revealed an edge energy of 8986.2 eV, which is shifted by ca. 2.5 eV to higher energies relative to the [Cu^I^(AN)]BF_4_ starting compound (8983.6 eV), confirming a divalent oxidation state for copper in **1**. Complex **1** is EPR silent in perpendicular mode at the X-band (Figure S3), consistent with its *S*_t_ = 1 ground state with considerable zero-field splitting; the signals shown in Figure S3A correspond to minor contributions (∼15% based on spin quantification; Figure S3B) arising from monomeric high- and low-spin Fe^III^ and Cu^II^ impurities.

### rRaman Spectroscopy

Confirmation for the presence of a peroxido moiety in **1** comes from rRaman measurements (in acetone/CH_3_CN 10:1 v/v solution at −90 °C upon excitation at 407 nm) as shown in Figure 1C. They exhibit a characteristic but very low-energy ν(O–O) vibrational mode at 739 cm^–1^ (^16^O_2_; 700 cm^–1^ with ^18^O_2_) and a diagnostic ν(Fe–O) mode at 635 cm^–1^ (^16^O_2_; 608 cm^–1^ with ^18^O_2_).^36^

### Theoretical Studies

**Figure 2:**
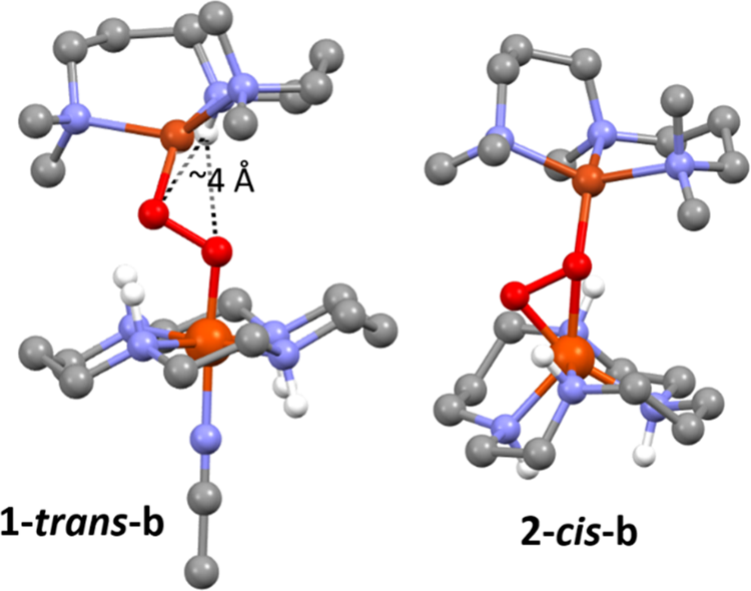
DFT-optimized structures **1-*****trans*****-b** (left) and **2-*****cis*****-b** (right); (color code: Cu brown, Fe orange, O red, N blue, C gray, H white (C–H protons are omitted for clarity)). See Tables S7 and S8, respectively, for their Cartesian coordinates.

For a better understanding of the actual peroxido binding mode in **1**, density functional theory (DFT) calculations were performed to identify optimized structures (Figure 2) and verify if they can theoretically reproduce the experimental results. Due to the flexibility of the mononucleating AN and cyclam ligands, a variety of different conformations and rotamers with and without an additional solvent molecule (CH_3_CN) binding to the Fe^III^ center were needed to be considered. These contain the cyclam ligand to Fe in either a folded (*cis*-V) conformation with alternating directions of the amino hydrogens (above vs below the ring) or a planar (*trans*-III) conformation with propylene-linked amino hydrogens pointing in the same and ethylene-linked ones in different directions.^37^ One of the axial binding sites to Fe in *trans*-cyclam is occupied by a coordinating solvent molecule CH_3_CN, while solvent coordination at the *cis* binding sites is sterically hindered by the bound [Cu(AN)(O_2_)] moiety. The AN ligand can adopt either a bent (**b**) or a planar (**p**) conformation. [(cyclam)Fe^III^(O_2_)Cu^II^(AN)]^3+^ starting structures with all ligand conformers, different O–O binding modes (μ-η^1^:η^1^, μ-η^1^:η^2^, μ-η^2^:η^1^, μ-η^2^:η^2^), and varying relative Fe–cyclam, O–O, and Cu–AN orientations were constructed and optimized. The same approach has been pursued for complex **2** (vide infra), in which the amino hydrogen of the AN ligand is replaced with a methyl group (MeAN). For each conformational combination, the lowest-energy structure, along with their relative energies, geometric parameters, Raman data of ^18^O-sensitive modes, and UV–vis absorption spectra are presented in Table S1.

All geometry optimizations yielded Cu^II^ in a distorted tetrahedral coordination environment, ligated by only one of the peroxido oxygens. Independent of the conformation of the Cu ligand, starting structures with *trans*-III-cyclam converged to a μ-η^1^:η^1^ (end-on) bridging motif with FeCu distances of 4.1–4.3 Å and a trend of short (1.8 and 1.9 Å, respectively) and long (2.6–2.8 Å) Fe···O and Cu···O distances, respectively. In contrast, those with *cis*-V-cyclam yielded a μ-η^2^:η^1^ motif with the peroxido unit binding side-on to Fe and end-on to Cu and shorter Fe···Cu distances of 3.7–3.9 Å, two shorter Fe···O distances (1.8 and 2.0 Å), and one short and one long Cu···O distances (2.0 and 2.9 Å, respectively). For both binding modes, the O···O distances of 1.43–1.45 Å are very similar. Broken-symmetry (BS) DFT suggests triplet ground states and thus ferromagnetic Fe^III^–Cu^II^ interactions for all of the structures.

**Figure 3:**
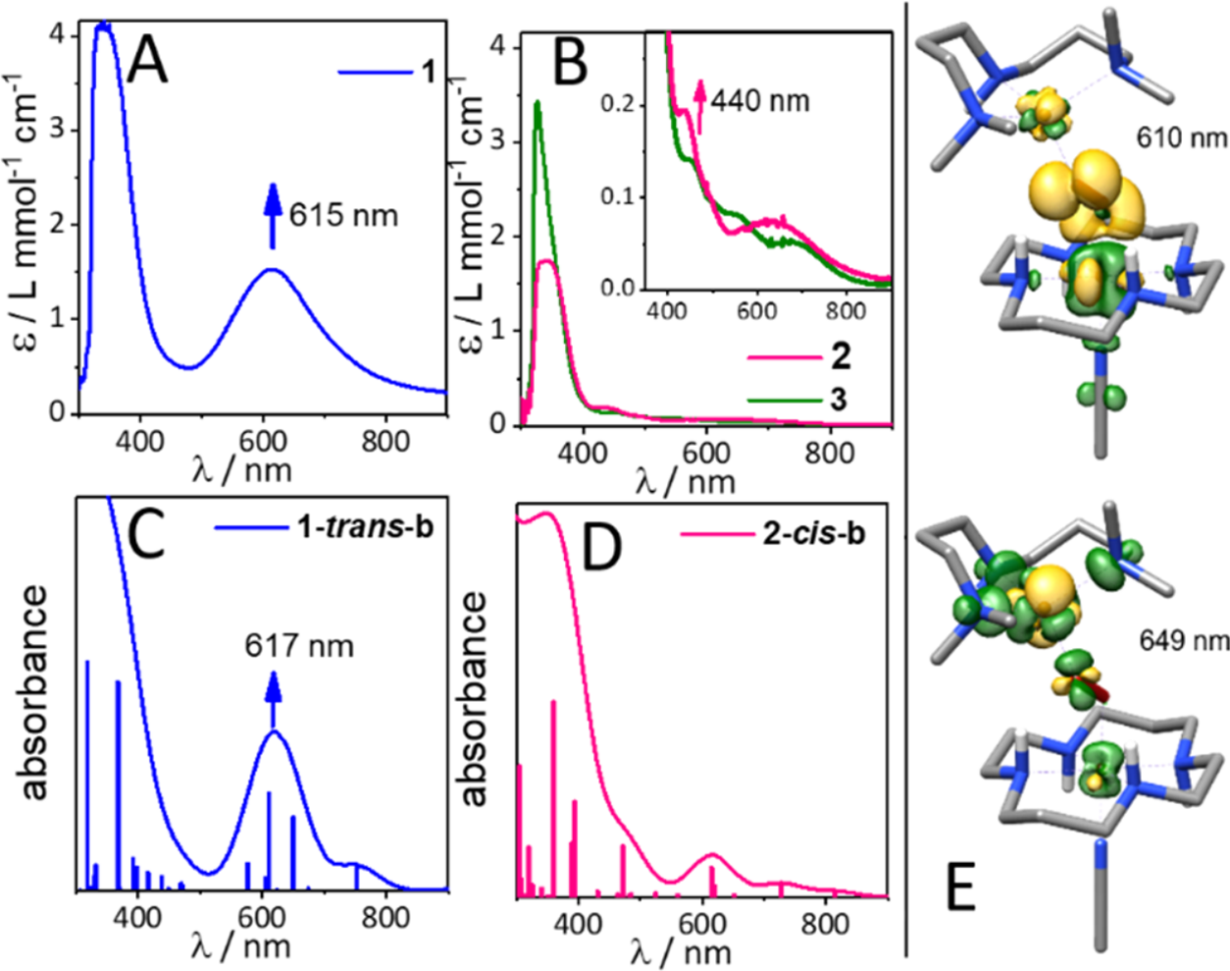
(A, B) Experimental UV–vis spectra of **1** and **2**, respectively. (C, D) Time-dependent DFT (TD-DFT)-based UV–vis absorption spectra for **1-*****trans*****-b** and **2-*****cis*****-b**, respectively, calculated from the individual transitions (sticks) by Gaussian broadening with 80 nm full width at half-maximum (fwhm). (E) Electron density difference maps (yellow: negative (loss) phase, green: positive (gain) phase, isovalue 0.0025 au) of the two main charge-transfer transitions at 610 and 649 nm in **1-*****trans*****-b**.

Most interestingly, depending on the O–O binding mode, the conformers can be divided into two groups according to both the calculated UV–vis spectra and the vibrational frequencies of the peroxido bridge (Table S1 and Figure 3). The Fe-cyclam conformation was found to be the controlling factor in determining the spectroscopic properties of the Fe^III^–O_2_–Cu^II^ core in **1**. While all models show strong absorption below 450 nm, only the *trans-*III-cyclam, i.e., the end-on μ-η^1^:η^1^ peroxido structures exhibit an intense absorption band centered in the range λ_LMCT_^cal^ = 600–640 nm (vide infra), whereas the *cis*-V-cyclam/μ-η^2^:η^1^ peroxido structures lack any strong absorption above 500 nm. At the same time, the end-on peroxido structures feature O–O stretching modes at 750–767 cm^–1^ (Δ_^16/18^O_^cal^ = −37 to −40 cm^–1^), while for the μ-η^2^:η^1^ peroxido structures, they are in the range 852–875 cm^–1^ (Δ_^16/18^O_^cal^ = −43 to −49 cm^–1^). Thus, models with a μ-η^1^:η^1^ peroxido motif generally reproduce the experimental results for **1** (λ_LMCT_ = 615 nm, ν(O–O) = 739 cm^–1^, Δ_^16/18^O_ = −40 cm^–1^). In particular, considering the respective lowest-energy structures (Figure 2; see Table S7), the calculated spectroscopic properties of **1-*****trans*****-b** (λ_LMCT_^cal^ = 617 nm, ν(O–O)^cal^ = 737 cm^–1^, and Δ_^16/18^O_^cal^ = −37 cm^–1^) are in excellent agreement with the experimental UV–vis and rRaman features of **1**. Thus, although the corresponding *cis* and *trans* models cannot be compared energetically due to the presence of an additional axial CH_3_CN ligand to the Fe^III^ ion in the *trans* structure, complex **1** can be safely assigned to an end-on μ-η^1^:η^1^ peroxido structure involving a [(CH_3_CN)(*trans*-III-cyclam)Fe^III^–O_2_–Cu^II^(*bent*-AN)]^3+^ (**1-*****trans*****-b**) structural motif as shown in Figure 2. This is also supported by the identification of another ^18^O-sensitive mode, involving the Fe–O stretching vibration in the range of 608–627 cm^–1^ (Δ_^16/18^O_^cal^ = −28 to −30 cm^–1^) for the end-on peroxido structures, in close proximity to the experimentally observed ^18^O-sensitive band at 635 cm^–1^ (Δ_^16/18^O_ = −27 cm^–1^) for **1**. The corresponding Fe–O vibration is calculated at 565–592 cm^–1^ (Δ_^16/18^O_^cal^ = −16 to −22 cm^–1^) for the μ-η^2^:η^1^ peroxido structures, which are significantly downshifted in energy relative to that of the experimentally observed value for **1**.

The absorption spectrum of **1** was also analyzed in detail. For the absorption band peaking at 617 nm, we find two main contributions at 610 and 649 nm in the calculated spectrum (Figure 3C). From the TD-DFT difference density (Figure 3E) for the electronic transition at 610 nm, we readily can identify it as a ligand-to-metal charge-transfer (LMCT) band from the peroxido bridge to the Fe^III^ ion. Closer inspection by means of the corresponding natural transition orbitals (NTOs, Figure S6) shows that it is characterized by two orbital pairs: mainly an O_2_^2–^ π*_v_ → Fe 3d_*xz*_ excitation (from the peroxido-Fe π-bonding MO into its π-antibonding counterpart, 65%), and a minor excitation (20%) from a mixed Fe 3d_*xy*_/Cu 3d_*z*^2^_ MO into a strongly delocalized σ-antibonding MO involving Fe 3d_*z*^2^_, O_2_^2–^ π_σ_, Cu 3d_*x*^2^–*y*^2^_ and orbitals on the N-donor atoms of both the AN and cyclam ligands. For the 649 nm band, the difference density and the NTOs indicate donor and acceptor orbitals delocalized mainly over the Cu ion, the peroxido ligand, and the nitrogens of the AN ligand. The donor orbital is composed of the Cu 3d_*z*^2^_ orbital with substantial admixture from the peroxido oxygens (π*_v_), while the acceptor orbital comprises the Cu 3d_*x*^*2*^*y*^*2*^_ and the σ-antibonding ligand orbitals from the AN nitrogens and peroxido oxygens (π*_σ_). Thus, in the presented nonheme Fe^III^–Cu^II^-peroxido species **1**, the specific O−O bridging motif is essential in defining both these transitions, such that a 600–650 nm band appears as a spectroscopic marker for this geometry.

### Synthesis and Characterization of **2**

An interesting structural aspect of **1-*****trans*****-b** is the orientation of the amino hydrogen of the AN ligand in bent conformation, which is placed at distances of 3.92 and 4.11 Å from the peroxido O_Cu_ and O_Fe_ atoms, respectively, and possibly involved in a weak secondary interaction (Figure 2, Scheme 3). Notably, the deuteration of the NH-group of AN resulted in an increased half-life of **1**, further supporting an interaction of the −NH group with the peroxido moiety in **1** (Figures S7–S8). However, this interaction is too weak to effect any significant change in the Fe–O or O–O vibration modes, as corroborated by theoretical (the Δ_^1/2^H_^cal^ isotope shift upon deuteration of the amino hydrogen is only 0.2 cm^–1^ for the O–O stretch and 0.01 cm^–1^ for the Fe–O stretch) and experimental rRaman (data not shown) studies. When the N–H group is replaced by a N–Me group, a μ-η^2^:η^1^ peroxido structure results in the corresponding [(*cis*-cyclam)Fe^III^(μ-η^2^:η^1^-O_2_)Cu^II^(MeAN)]^3+^ complex **2**. Experimentally, the iron center in **2** is also low-spin Fe^III^ as evident from zero-field Mößbauer studies (δ = 0.24 mm s^–1^ and Δ*E*_Q_ = −2.51 mm s^–1^), but unlike the *S*_t_ = 1 ground state in **1**, a *S*_t_ = 0 assignment is deduced for **2** based on the applied field Mößbauer, which is also consistent with EPR studies (Figures S9 and S10). The different peroxido binding motifs and coupling situations in **1** and **2** are reflected in their different spectroscopic properties (Figure 3A/B). Complex **2** lacks any UV–vis absorption feature above 400 nm and exhibits a higher peroxidic stretching frequency, ν(O–O) = 856 cm^–1^ (^16^O_2_; 803 cm^–1^ for ^18^O_2_, Figure S11), relative to that of **1** (ν(O–O) = 739 cm^–1^). Our theoretical studies predict a **2-*****cis*****-b** structure with a μ-η^2^:η^1^ peroxido binding motif (Figure 2, Table S8), which can reproduce the experimentally observed UV–vis absorption and the O–O stretching modes in **2** (Figure 3 and Table S1).

### Extended X-ray Absorption Fine Structure (EXAFS) Analysis of **1** and **2**

Analysis of the extended X-ray absorption fine structure (EXAFS) at the Fe and Cu K-edges of **1** and **2** (Figures S4, S5, Tables S2–S4) is in reasonable agreement with the bond lengths calculated by DFT for **1-*****trans*****-b** and **2-*****cis*****-b**. Satisfactory EXAFS simulations of the iron spectra of **1**/**2** were obtained with 4 N scatterers at a distance of 1.99 Å and additional O contributions at (mean) shorter (1.79/1.73 Å) and longer (2.68/2.12 Å) distances to the iron center that can be ascribed to the O atoms of the end-on peroxido unit in **1** (DFT: Fe–O1: 1.78 Å; Fe–O2: 2.72 Å) or the side-on peroxido unit in **2** (DFT: Fe–O1: 1.78 Å, Fe–O2: 1.99 Å). The apparent shortest Fe–O bonds in **1** and **2** from the EXAFS simulations may in part reflect contributions of Fe^IV^=O species in the samples (see below). Also, for the copper spectra, the simulations revealed two O atoms at 1.87/1.84 and 2.57/2.55 Å in **1**/**2**, respectively (DFT: Cu–O2: 1.91/2.00 Å, Cu–O1: 2.62/2.93 Å) (see Tables S2–S4 for details).^38^ We note that in the presence of multiple species in the solution samples of the complexes, perfect agreement between interatomic distances from XAS and DFT is not expected, as a limited number of variable parameters (e.g., bond lengths) have to be used in EXAFS fit analysis to avoid data overinterpretation. Furthermore, low concentrations of **1** and **2** and the presence of scatterers from the ligand scaffold in similar distances did not allow for a reliable determination of Fe···Cu distances above 3.5 Å via EXAFS.

### Axial Ligand Effect on the Peroxido Binding Motif

**Scheme 2:**
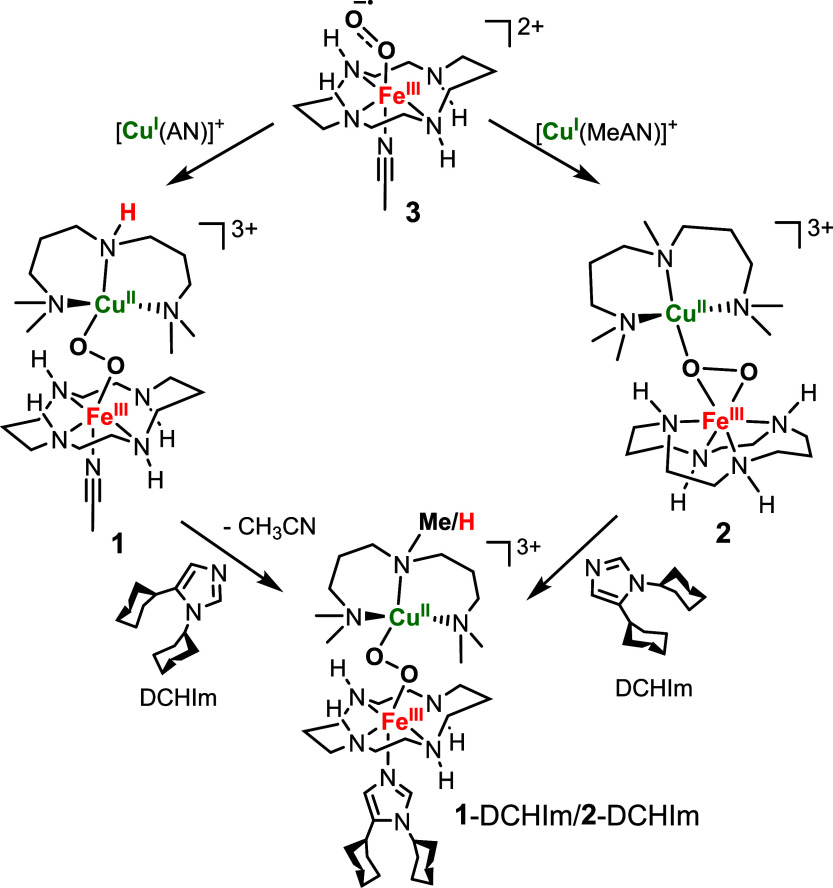
Overview of the Discussed Complexes and Intermediates and Their Reactivity

**Figure 4:**
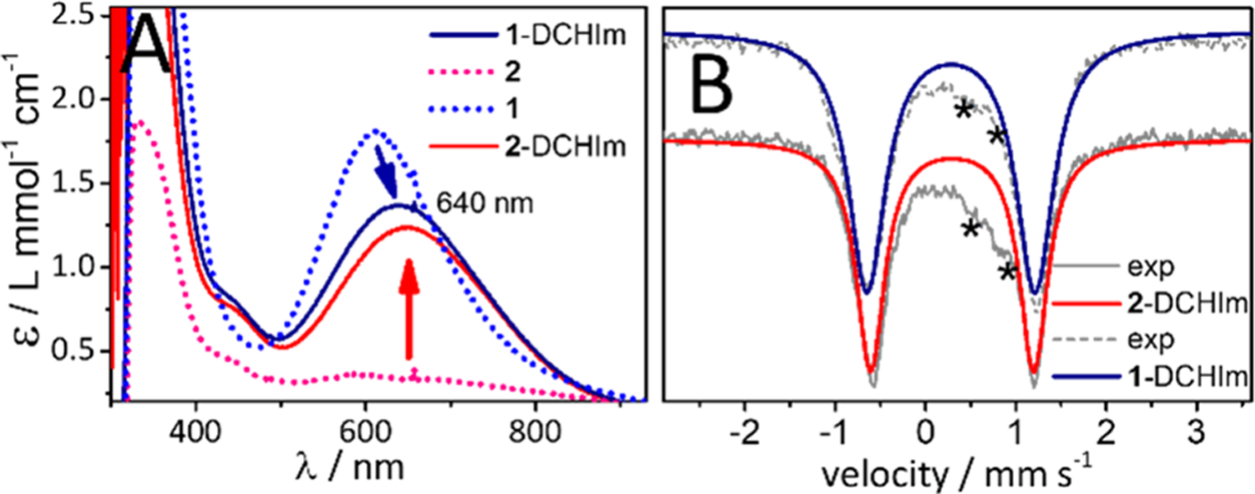
(A) UV–vis absorption spectral changes associated with the direct addition of DCHIm to **1** (dashed blue) and **2** (dashed pink) to form **1**-DCHIm (dark blue) and **2**-DCHIm (red), respectively (acetone/CH_3_CN 10:1 v/v mixture, −90 °C). (B) Zero-field Mößbauer spectra of **1**-DCHIm (gray-dashed: experimental, dark blue: simulated main species) and **2**-DCHIm (gray: experimental, red: simulated main species). Minor species are marked by asterisks, see Table S6 and Figure S12 for details.

As suggested by DFT, the binding of an axial CH_3_CN ligand triggers the isomerization of the *cis*-cyclam moiety in **2** to *trans*-cyclam in **1**, with the concomitant change of the peroxido binding motif at Fe from side-on to end-on (Scheme 2 and Figure 2). Consistent with this suggestion, the addition of 1,5-dicyclohexylimidazole (DCHIm) to a CH_3_CN/acetone solution of **2** at −90 °C led to the generation of a blue–green species **2**-DCHIm (*t*_1/2_ = 40 s at −90 °C) that shows the characteristic low-energy absorption feature at 640 nm (Figure 4A) associated with the μ-η^1^:η^1^ peroxido binding motif. The axial CH_3_CN ligand in **1** can also be replaced by DCHIm as evidenced by the red shift of the 615 nm band in **1** to 640 nm in **1**-DCHIm (*t*_1/2_ = 60 s at −90 °C). The Fe^III^ centers in **1**-DCHIm and **2**-DCHIm are electronically very similar as evidenced from their near identical Mößbauer parameters: for **1**-DCHIm, δ = 0.28 mm s^–1^ and |Δ*E*_Q_| = 1.87 mm s^–1^, and for **2**-DCHIm, δ = 0.29 mm s^–1^ and |Δ*E*_Q_| = 1.81 mm s^–1^ (Figure 4B, Figure S12 and Table S6). Both are silent in conventional X-band EPR measurements in perpendicular mode indicating an integer total spin state (Figure S13). Although the metastable nature of **1**-DCHIm and **2**-DCHIm prevented us from measuring their ν(O–O) vibration modes, based on the similarity in their absorption and Mößbauer features, the presence of a [(DCHIm)(*trans*-cyclam)Fe^III^(μ-η^1^:η^1^-O_2_)Cu^II^(AN/MeAN)]^3+^ motif can safely be concluded in both the complexes.

### Reactivity Studies of **1** versus **2** with PhOH

**Scheme 3:**
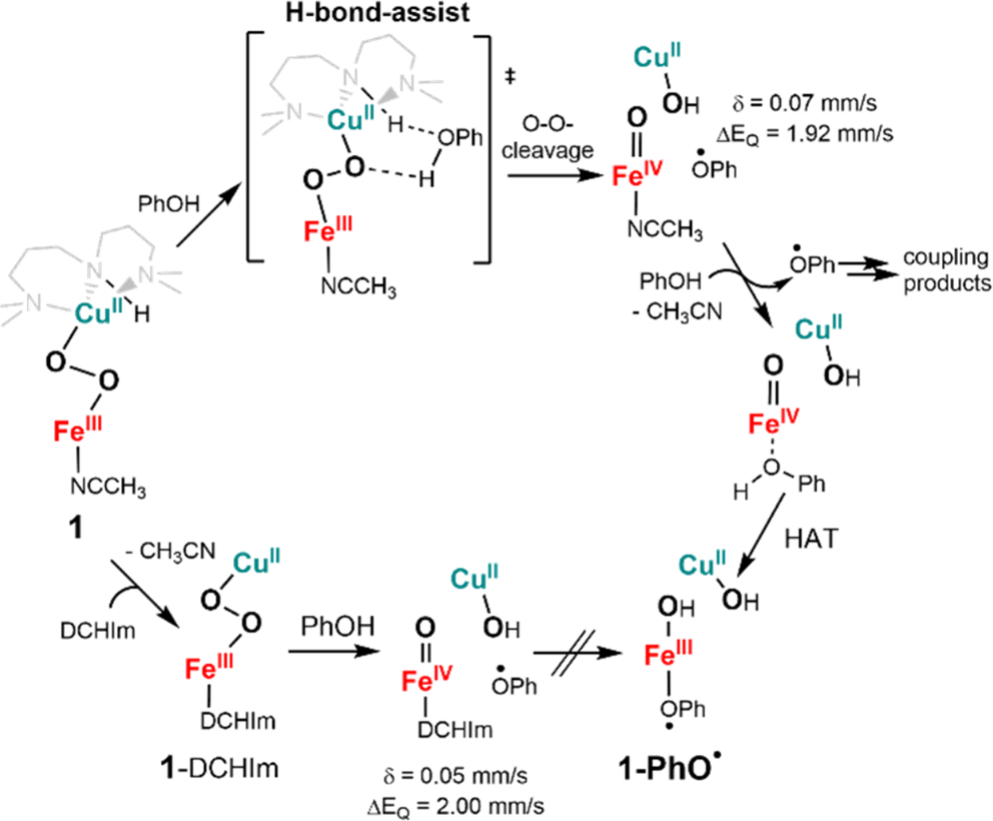
Proposed Mechanism for the Reaction of **1** and 1-DCHIm with Phenol via a H-Bond-Assisted Mechanism

**Figure 5:**
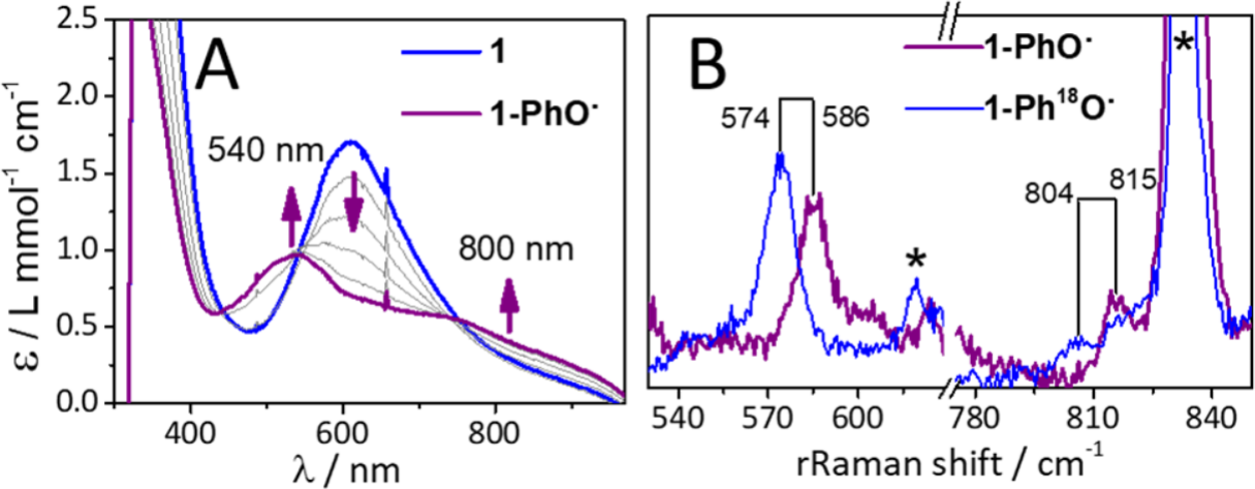
(A) UV–vis spectral changes associated with the conversion of **1** (blue) into **1-PhO**^•^ (purple) upon addition of 5 equiv of phenol (acetone/CH_3_CN 10:1 v/v mixture, −90 °C). (B) rRaman spectra of **1-PhO^•^** (purple) and **1-Ph**^**18**^**O^•^** (blue) measured with 568 nm excitation (2 mW) in an acetone-*d*_6_/CD_3_CN 10:1 v/v solution. Solvent features are marked by asterisks.

The different electronic structures of **1** and **2** are also reflected in their reactivity properties. For example, complex **1**, and not complex **2**, exhibits reactions with phenol, possibly stressing the importance of the *S*_t_ = 1 ground state and the weak –NH secondary interaction in the hydrogen atom transfer (HAT) reactivity of the peroxido complexes. Addition of phenol to **1** showed a fast reaction that could be monitored by UV–vis (Figure 5A). The typical absorption feature of **1** at 615 nm vanished, while a new purple species **1-PhO^•^** is observed with an absorption maximum at 540 nm (ε = 1.05 L mmol^–1^ cm^–1^) and a broad feature between 600 and 1000 nm. When substituted phenols (*p*-kresol, *p*-methoxyphenol, and *p*-chlorophenol) were added to **1**, a shift of the absorption maximum (**1-**^***p*****-Me**^**PhO^•^**: λ_max_ = 525 nm, **1-**^***p*****-OMe**^**PhO^•^**: λ_max_ = 580 nm, and **1-**^***p*****-Cl**^**PhO^•^**: λ_max_ = 570 nm) can be observed, corroborating the binding of phenol-derived species (Figure S14). Gas chromatography-mass spectrometry (GC-MS) analyses of the reaction mixtures at room temperature revealed the formation of coupled phenol products (4,4′-bis(2,6-di*-tert*-butylphenol) (19%), 3,3′,5,5′-tetra-*tert*-butyldiphenoquinone (10%), and 2,6-di-*tert*-butyl-1,4-benzoquinone (10%) for reaction with 2,6-di*-tert*-butylphenol and 5,5′-dimethoxy[1,1′-biphenyl]-2,2′-diol (∼20%) for reaction with 4-methoxyphenol) in moderate to high yields (Figures S15, S16 and Table S5). Further spectroscopic investigations show that the oxidative coupling of the phenol is accompanied by the reductive cleavage of the peroxido bridge in **1** and the formation of copper(II) and iron(III)-phenoxyl radical species. The initially formed oxoiron(IV) species presumably reacts with excess PhOH to generate the iron(III)-phenoxyl radical species (Scheme 3). Indeed, the Mößbauer spectrum of **1-PhO^•^** (Figure S17) shows the formation of a major Fe^III^ low-spin species in 70% yield (δ = 0.25 mm s^–1^, |Δ*E*_Q_| = 2.57 mm s^–1^) and a residual Fe^IV^ (δ = 0.07 mm s^–1^, |Δ*E*_Q_| = 1.92 mm s^–1^) species in 25% yield. Notably, Mößbauer parameters of the Fe^IV^ species are comparable to the parameters for an authentic [Fe^IV^(O)(*trans*-cyclam)(CH_3_CN)]^2+^ reported previously (δ = 0.05 mm s^–1^, Δ*E*_Q_ = 2.49 mm s^–1^, Table S6);^34^ the differences in δ and Δ*E*_Q_ values are plausibly attributed to additional interactions from the Cu^II^, PhO^•^, or OH moieties (see Scheme 3). The EPR spectrum reveals the formation of multiple overlapping *S* = 1/2 species (Figure S18) arising from Cu^II^ centers in near-quantitative yields. rRaman studies were performed (Figures 5B and S19) to confirm the **1-**^**X**^**PhO**^•^ assignment. Two features were observed for **1-PhO^•^** at 815 and 586 cm^–1^, which showed no shift when ^18^O labeled **1** was used in the reaction, but shifted to 804 and 574 cm^–1^, respectively, when ^18^O-labeled PhOH was used. The positions of these bands were also found to be sensitive to the nature of the *para*-substituents (Figure S19). Based on a recent study where we spectroscopically characterized a phenoxyl radical bound to an Fe^II^ center^39^ and previous reports of metal bound phenoxyl radicals,^40,41^ we assign the 586 and 815 cm^–1^ bands to Fe–O and in-plane phenyl ring bending vibrations, respectively, for a metal bound PhO^•^ radical.

The *S*_t_ = 1 [(CH_3_CN)(*trans*-cyclam)Fe^III^(μ-η^1^:η^1^-O_2_)Cu^II^(AN)]^3+^ core in **1** thus undergoes a reductive O–O bond cleavage step via a net H-atom transfer from phenol to generate [(AN)Cu^II^–OH]^+^, [Fe^IV^(O)(*trans*-cyclam)(CH_3_CN)]^2+^ and PhO^•^ (Scheme 3). A previous study on a heme Fe^III^–O_2_–Cu^II^ species^39^ has shown that the phenol can facilitate O–O cleavage via two possible mechanisms, which differ by the amount of proton transfer prior to the transition state. One mechanism involves nearly complete proton transfer from the phenol to the peroxo before the barrier. The second mechanism involves O–O homolysis by phenol H-bonding to peroxo, with the proton transfer occurring after the barrier. The two mechanisms can be distinguished by kinetic isotope effect (KIE) data for an H-atom-donating phenol inducing O–O cleavage. A KIE of 1.43 has been determined for the O–O bond cleavage step (Figure S20) in **1**, consistent with the hydrogen-bonded mechanism (Scheme 3);^42^ a much higher KIE (>5) is expected for complete proton transfer before the barrier. This H-bond assistance is further supported by an observed secondary KIE of 1.20 (Figure S20) in the phenol reaction upon deuteration of the –NH group of the AN ligand in **1**. An increase of the reaction rate for the formation of **1-**^**X**^**PhO^•^** with the p*K*_a_ of the corresponding substituted phenols (*k*_2_ = 0.38 for **1-**^***p*****-Me**^**PhO^•^**, *k*_2_ = 0.83 for **1-PhO^•^**, *k*_2_ = 1.53 for **1-**^***p*****-Cl**^**PhO^•^**, Figure S21) is also consistent with the mechanism shown in Scheme 3.

### Effect of the Axial Ligand on PhOH Reactivity of **1**

The complex [Fe^IV^(O)(*trans*-cyclam)(CH_3_CN)]^2+^ undergoes a side reaction with excess PhOH to generate [(*trans*-cyclam)Fe^III^(PhO^•^)(OH)]^2+^. This reaction is presumably triggered by the replacement of the axial CH_3_CN ligand in [Fe^IV^(O)(*trans*-cyclam) (CH_3_CN)]^2+^ by PhOH, followed by HAT by the Fe^IV^=O center. Indeed, the side reaction is completely stopped in the reaction of **1**-DCHIm with PhOH, where replacement of the stronger axial ligand DCHIm by PhOH is not favored, and the Mößbauer analysis of the reaction mixture shows the formation of [Fe^IV^(O)(*trans*-cyclam)(DCHIm)]^2+^ in near-quantitative yields (δ = 0.05 mm s^–1^, |Δ*E*_Q_| = 2.00 mm s^–1^, Table S6 and Figures S22–S24). Consistent with our presumption, an authentic sample of [(DCHIm)(*trans*-cyclam)Fe^IV^=O]^2+^ obtained by replacement of the CH_3_CN ligand in [(CH_3_CN)(*trans*-cyclam)Fe^IV^=O]^2+^ by DCHIm at −50 °C did not show any reactivity with PhOH (Figure S24). Notably, both **1**-DCHIm and **2**-DCHIm show increased yields of the coupled phenol products compared to **1** (Table S5 and Figure S25), reflecting the release of the phenoxyl radical instead of coupling to the iron center.

### Reactions of **1** and **2** with PPh_3_

Intermediate **1** also reacts at −90 °C with PPh_3_ forming OPPh_3_ (identified by GC-MS, Figure S26) and a new species **1-Cu** with a UV–vis absorption feature at 675 nm (ε = 0.19 L mmol^–1^ cm^–1^) that further decays above −50 °C (Figure S27). Notably, both **2** and **3** also reacted with PPh_3_, albeit with rates,respectively, 1 and 3 orders of magnitude slower than **1** (Figure S28–S30). This confirms that the O–O bond in **1** is more activated relative to that in **2** and **3**. In particular, the higher reaction rates of **1** and **2**, relative to **3**, demonstrate the positive influence of Cu in the reductive O–O bond cleavage reaction. The absorption spectrum and Mößbauer parameters (Figure S31, δ = 0.07 mm s^–1^, |Δ*E*_Q_| = 2.08 mm s^–1^) of **1-Cu** are similar compared to that reported for [Fe^IV^(O)(*trans*-cyclam)(CH_3_CN)]OTf_2_ (Table S6).^34^ The XANES spectra at the Fe K-edge of **1-Cu** and **2-Cu** (Figure S32) show an intense pre-edge peak at about 7115 eV that is typical for oxoiron complexes. EXAFS analysis additionally revealed a short iron–oxygen bond of 1.62 Å in ca. 50% of the complexes (Figure S33, Table S3), which is indicative of an Fe^IV^=O species. The ESI-MS spectrum of **1**-**Cu** shows a signal at *m*/*z* = 706.1 with mass and isotope distribution pattern consistent with a [Fe(O)(cyclam)(OTf)Cu(AN)(Cl)]^+^ assignment, which is shifted by two and one mass units upon ^18^O- and ^57^Fe-labeling, respectively (Figure S33). **1-Cu** is EPR silent in perpendicular mode X-band EPR (Figure S34). All of these point to the formation of Fe^IV^=O, Cu^I^, and PPh_3_O products in the reaction of the *S*_t_ = 1 [(CH_3_CN)(*trans*-cyclam)Fe^III^(μ-η^1^:η^1^-O_2_)Cu^II^(AN)]^3+^ (**1**) with PPh_3_.

## Conclusions

In summary, a nonheme iron(III)-superoxo complex **3** reacts with [Cu^I^(AN)] generating a low-spin iron(III)-peroxido-copper(II) complex **1** with a *S*_t_ = 1 ground state. **1** undergoes efficient O–O bond cleavage in the presence of H-bonded PhOH and a strong axial ligand DCHIm leading to the stoichiometric formation of an oxoiron(IV) species. This process has some relevance to the CcO catalytic cycle. A key matter that remains unsolved in CcO is the reaction coordinate connecting intermediates **A** and **P**_**M**_. While it has not been observed experimentally, the involvement of a peroxido level intermediate (**I**_**P**_ in Scheme 1) in CcO has been suggested in many studies, whereby a *S*_t_ = 1 [Fe^III^(μ-η^1^:η^1^-O_2_)Cu^II^] core undergoes a fast low-barrier (<12.4 kcal mol^–1^) O–O bond cleavage via H-atom abstraction from Tyr-OH that is involved in a hydrogen-bonding network with water.^5,43−45^ In the present study, complex **1** not only reproduces the proposed *S*_t_ = 1 [Fe^III^(μ-η^1^:η^1^-O_2_)Cu^II^] motif, suggested for **I**_**P**_, but the −NH group of the AN ligand also provides the platform for HAT from a H-bonded phenol that is required for the efficient O−O bond cleavage step. The fast and efficient O–O cleavage occurring in **1** can be attributed to the observed ferromagnetic coupling between the low-spin Fe^III^ and Cu^II^ centers in **1**, which, as previously proposed based on theoretical studies^16−19^ in **I**_**p**_ ensures efficient reductive O–O rupture to form *S* = 1 Fe^IV^=O and antiferromagnetically coupled Cu^II^ and PhO^•^ via electron transfer from PhO^–^ without the necessity of any spin-surface crossing. However, the exchange-coupled Cu^II^–PhO^•^ species is presumably transient in our case and decays to mononuclear Cu^II^ (detected in EPR, data not shown) and coupled phenol products. Consistent with this proposition, a related low-spin Fe^III^-peroxido-Cu^II^ species **2** in otherwise similar Fe- and Cu-coordination environments, but with an *S*_t_ = 0 ground state due to antiferromagnetic coupling between the Fe^III^ and Cu^II^ centers, did not show any reactivity with PhOH.

Complexes **1** and **2** differ in their peroxido binding mode to Fe^III^ (end-on vs μ-η^2^:η^1^ in **1** and **2**, respectively), which is also reflected in their ν(O–O) modes and the transitions of the O_2_^2–^ to Fe^III^ charge transfer in the UV–vis absorption spectrum. Considering the substantial differences in structural parameters for the two peroxido bridging motifs and of the Fe coordination geometries, it is apparent that metal–ligand orbital interactions and MO energies will be largely different in **1** and **2**. The fact that in **2**, the Fe^III^ ion is directly ligated by both instead of only one O donor leads to a considerably different interaction of the peroxo π* with the Fe 3d orbitals. For example, the π*_v_ orbital exhibits stronger interactions with the Fe 3d_*xy*_ and 3d_*yz*_ orbitals at the expense of the interaction with its 3d_*xz*_ orbital compared to the situation in complex **1**. It can thus be understood that the dominant excitation within the strongest LMCT transition (610 nm) in **1** does not exist as such in **2** due to a mitigated π-bonding interaction between the O_2_^2–^ π*_v_ and the Fe 3d_*xz*_ orbital.

Comparison of **1** to the very well characterized heme-based iron-peroxo-copper adduct [(DCHIm)(F_8_)Fe–O_2_–Cu(AN)]^+^ by Karlin and Solomon,^12,28,42^ which they have identified to possess a μ-η^1^:η^1^/end-on binding mode, shows that they resemble each other in that they both feature similar LMCT excitations from the O_2_^2–^ π*_v_ orbital into the Fe 3d_*xz*_ and Cu 3d_*x*^*2*^*–y*^*2*^_ orbitals, albeit at significantly lower energies (789 and 951 nm, respectively) and of lower intensity. As orbitals from the equatorial ligands to the Fe^III^ ions contribute only insignificantly in both cases, comparison between the heme and nonheme complexes is legitimate in this respect. The higher energy of the transition from the π-bonding O_2_^2–^ π*_v_/Fe 3d_*xz*_ MO into its π-antibonding counterpart in **1** than in [(DCHIm)(F_8_)Fe–O_2_–Cu(AN)]^+^ indicates a stronger Fe–O bond, in line with the somewhat shorter calculated Fe···O distance (1.79 in **1** vs 1.82 Å in [(DCHIm)(F_8_)Fe–O_2_–Cu(AN)]^+^). At the same time, the considerably lower ν(O–O) stretching vibrational energy of 739 cm^–1^ in **1** compared to 794 cm^–1^ in the heme complex (Table 1) demonstrates concomitant weakening of the O–O bond, here consistent with the longer O···O distance (1.45 in **1** vs 1.40 Å in [(DCHIm)(F_8_)Fe–O_2_–Cu(AN)]^+^).

**Table 1:** Comparison of the Frequencies of the O–O Stretching Modes and Selected Bond Lengths of Fe–O_2_–Cu Intermediates

compound	ν(O–O) [Δ_^16/18^O_] (cm^–1^)	O···O (Å)	Fe···Cu (Å)
**1**	739 [−40]	nd	nd
**1-*****trans*****-b** (DFT; end-on)	735 [−39]	1.45	4.21
**2**	856 [−50]	nd	nd
**2-*****cis*****-b** (DFT; μ-η^2^:η^1^-O_2_)	862 [−49]	1.43	3.71
[F_8_Fe–O_2_–CuAN]^+^ (side-on)^28^	756 [−48] (DFT 821)	1.46 (DFT)	3.63 (XAS) 3.73 (DFT)
[(TMP)Fe–O_2_–Cu(5MeTPA)]^+^ (μ-η^2^:η^1^-O_2_)^22^	790 [−44]	1.46 (XRD)	3.92 (XRD)
[(DCHIm)F_8_Fe–O_2_–CuAN]^+^ (end-on)^28^	796 [−42] (DFT 840)	1.40 (DFT)	4.01 (XAS) 4.01 (DFT)
resting oxidized state of CcO^5^	750^a^	1.49–1.70	4.6–4.8

a Alternatively assigned as an O-sensitive histidine breathing mode.^33^

In heme-copper model chemistry, a lower value for ν(O–O) is in general correlated with a side-on binding mode to one or both metal centers. Table 1 shows selected examples of heme-copper model complexes (note that the [(F_8_)Fe–O_2_–Cu(AN)] system also incorporates the AN ligand at the copper site) with a linear correlation between the observed O–O stretching frequency and the Fe···Cu-distance, corresponding to the assignment from side-on^11,13,24^ to end-on^11,27,28^ via a μ-η^2^:η^1^-(O_2_)^22,27^ binding mode.^5,12^ Similar observations and assignments were also made for other heme-based models. Studies on the resting oxidized states of CcOs show a more complex situation.^1,29−33^ While the investigated structures and trapped intermediate states are not directly involved in the catalytic cycle of CcO, they are thought to provide valuable insights into the character of actual intermediate **I**_**P**_. All studies agree on a long Fe···Cu distance of about 4.6–4.8 Å, which would only allow for an end-on bound peroxido unit, but the O–O-bond length could not be determined precisely.^5^ The O–O frequency that is of course correlated with the bond length is also a matter of discussion. In initial studies, a rRaman feature at 750 cm^–1^ was observed in one resting oxidized CcO example and assigned to the O–O stretching mode,^7,32^ but a recent study discussed this feature as the wagging of the axial histidine that is sensitive to the peroxido unit.^33^ One reason why the assignment of the 750 cm^–1^ band to the O–O mode was questioned was the discrepancy between its low energy, indicating a side-on binding mode in heme chemistry while the environment in the active center would presuppose an end-on bound O_2_^2–^. The present report of the *S*_t_ = 1 [(CH_3_CN)(*trans*-cyclam)Fe^III^(μ-η^1^:η^1^-O_2_)Cu^II^(AN)]^3+^ complex **1**, which can reproduce both the UV–vis absorption and rRaman features associated with the as-isolated CcO, corroborates that a bridging peroxido moiety in an end-on bound O_2_^2–^ structure and with a significantly weak O–O bond can be accommodated within the Fe···Cu distance of >4.0 Å that is established in CcO. Whether the 750 cm^–1^ signal in the resting state of CcO can be possibly assigned as the O–O-vibration mode associated with an end-on Fe^III^–O_2_–Cu^II^ peroxido binding motif is now an intriguing question, which needs further investigation.

## Supporting Information Available

Instrumental and physical methods, synthesis of compounds and intermediates, reactivity and kinetic data, theoretical studies, Figures S1–S34 (further characterization and reactivity data), and Tables S1–S8 (reactivity and DFT data) (PDF)
