# Supplementary material for: A Bioinspired Nonheme FeIII–(O22–)–CuII Complex with an St = 1 Ground State
Source: J Am Chem Soc. 2024 Jul 5;146(36):24808–17. doi: 10.1021/jacs.4c04492 (PMC11403606; doi:10.1021/jacs.4c04492)
Supplement: Supplementary file 1 — ja4c04492_si_001.pdf [file ja4c04492_si_001.pdf]

Supporting Information for

A Bioinspired Non-heme Fe^{III}−(O_2^{2-})−Cu^{II} Complex with a S_t = 1 Ground State

Dustin Kass,[ǀ] Sagie Katz,[ꜱ] Hivda Özgen,[ǀ] Stefan Mebs,[†] Michael Haumann,[†] Ricardo García-Serres,[ψ] Holger Dau,[†] Peter Hildebrandt,[ꜱ] Thomas Lohmiller,[ǀ,¶,]* Kallol Ray[ǀ,]*

[ǀ]Institut für Chemie, Humboldt-Universität zu Berlin, Brook-Taylor-Straße 2, 12489 Berlin, Germany
[ꜱ] Department of Chemistry, Technische Universität Berlin, Straβe des 17. Juni 135, 10623 Berlin, Germany
[†] Department of Physics, Freie Universität Berlin, Arnimallee 14, 14195 Berlin, Germany
[ψ]Université Grenoble Alpes, CEA, CNRS, Laboratoire de Chimie et Biologie des Métaux, 38000 Grenoble, France
[¶]EPR4Energy Joint Lab, Department Spins in Energy Conversion and Quantum Information Science, Helmholtz-Zentrum Berlin für Materialien und Energie GmbH, Albert-Einstein-Straße 16, 12489 Berlin, Germany

*Corresponding author(s) email: kallol.ray@chemie.hu-berlin.de; thomas.lohmiller@hu-berlin.de

Content

1. Experimental section ... 3
   1.1. Materials ... 3
   1.2. Instrumentation and experimental methods ... 3
   1.3. Synthesis of compounds and intermediates ... 4
      1.3.1. Synthesis [Cu(MeAN)]BF_4, [Cu(AN)]BF_4 and [Cu(AN-D)]BF_4 ... 4
      1.3.2. Generation of intermediates **1**, **2** and **3** and their ^{57}Fe- and ^{18}O-enriched variants ... 4
      1.3.3. Generation of **1-$^{p\text{-}X}$PhO•**, **1**-DCHIm and **2**-DCHIm ... 5
   1.4. Reactivity studies and determination of rate constants ... 5
2. Theoretical section ... 6
3. Supplementary Tables and Figures ... 7
   3.1. Supplementary Figures S1-34 ... 7
   3.2. Supplementary Tables S1-S8 ... 22
4. References ... 29

1. Experimental section

1.1. Materials

The chemicals employed were purchased from the companies ABCR, ACROS, SIGMA-ALDRICH and TCI, and used without further purification. Anhydrous solvents (acetonitrile, diethylether, dichloromethane) were purchased from CARL-ROTH GmbH under the tradename ROTIDRY (>99.5%, < 50 ppm H_2O) and degassed by freeze-pump-thaw methods and dried over activated molecular sieve (3 Å or 4 Å) prior to use. Dried and degassed acetone was purchased from THERMO SCIENTIFIC under the trade name ACRO SEAL (>99.8%, extra dry). Deuterated solvents and $^{18}O_2$ were purchased from EURISOTOP. Preparation and handling of air or water sensitive compounds was performed under an inert atmosphere using either Schlenk techniques or a glovebox GS111717 from GS GLOVEBOX SYSTEMTECHNIK filled with N_2. Nitrogen and Argon of quality 5.0 were used for this purpose and were purchased from AIR LIQUIDE.

1.2. Instrumentation and experimental methods

Electrospray ionization mass spectrometry. ESI-MS spectra of organic molecules and inorganic complexes in solution were recorded by using an ADVION EXPRESSION CMS spectrometer (in *typical* ionization mode); acetonitrile was used as an eluent. Thermally unstable complexes were directly injected from the freshly thawed solution. The analysis of the data was carried out with the ADVION DATA EXPRESS Version 6.0.11.3.

Gas chromatography. GC analysis was carried out using an AGILENT 7890B gas chromatograph (HP5 column, 30 m) with a flame-ionization detector coupled to an EI-MS AGILENT 5977B spectrometer with a triple-axis detector. The instrument was equipped with an autoinjector AGILENT G4513A (injection of approx. 10 µL).
The GC method starts with an oven temperature of 75 °C (hold time 0.5 min) and includes two ramps (ramp 1: 75 °C-190 °C, 10 °C/min; ramp 2: 190 °C-300 °C, 20 °C/min, hold time 10 min at 300 °C), a total run-time of 27.5 min and a solvent delay of 2.9 min. MS peaks were analyzed and compared with the library database of NIST MS Search 2.3. All EI-MS spectra of the detected peaks were in good agreement with the library database of the expected substances.

Mößbauer spectroscopy. Mößbauer spectra in the absence of magnetic field were recorded on a SEECO MS6 spectrometer that comprises the following instruments: a JANIS CCS-850 cryostat, including a CTICRYOGENICS closed cycle 10 K refrigerator, and a CTI-CRYOGENICS 8200 helium compressor. The cold head and sample mount are equipped with calibrated DT-670-Cu-1.4L silicon diode temperature probes and heaters. Temperature is controlled by a LAKESHORE 335 temperature controller. Spectra are recorded using a LND-45431 Kr gas proportional counter with beryllium window connected to the SEECO W204 γ-ray spectrometer that includes a high voltage supply, a 10 bit and 5 µs ADC and two single channel analyzers. Motor control and recording of spectra is taken care of by the W304 resonant γ-ray spectrometer. For the reported spectra a RIVERTEC MCO7.114 source (^{57}Co in Rh matrix) with an activity of about 1 GBq was used. All spectra were recorded in a plastic sample holder with a frozen solution sample or as a solid (if noted) at ~15 K. Data were accumulated for about 12 to 24 hours and spectra were simulated using WMOSS4F.[1]
Applied-field Mößbauer spectra were measured on an OXFORD INSTRUMENTS Spectromag 4000 cryostat containing an 8 T split-pair superconducting magnet. The spectrometer was operated in constant acceleration mode in transmission geometry, with the magnetic field applied parallel to the direction of the gamma rays. The isomer shifts are referenced against a room temperature metallic iron foil. Analysis of the data was performed using the in-house developed Python package easyMoss.[2] All spectra were modelled with slow relaxation.

X-ray absorption spectroscopy. XAS at the Fe-*k*-edge was performed at beamline KMC-3 at the BESSY-II synchrotron (Helmholtz Center Berlin, Germany) as described earlier[3] using a set-up including a Si[111] double-crystal monochromator, a 13-element energy-resolving Si-drift detector (RaySpec) for X-ray fluorescence monitoring, and DXP-XMAP pulse-processing electronics (XIA). Samples were held at 20 K in a liquid-helium cryostat (Oxford). The energy axis of the monochromator was calibrated (accuracy ±0.1 eV) using the K-edge spectra of iron or copper metal foils (fitted reference energies of 7112 eV or 8979 eV in the first derivative spectra). The spot size on the samples was ca. 1.5 x 3.0 mm (vertical x horizontal) as set by a focusing mirror and slits. X-ray fluorescence spectra were collected using a continuous scan mode of the monochromator (scan duration ~10 min). Up to 6 scans were averaged (1-2 scans per sample spot) for signal-to-noise ratio improvement. XAS data were processed (dead-time correction, background subtraction, normalization) to yield XANES and EXAFS spectra using our earlier described procedures and in-house software. K-edge energies were determined at half-height (50 % level of normalized absorption). k^3-weighted EXAFS spectra were simulated with in-house software and phase functions from FEFF9 (S_0^2 was 0.8 for Fe and 1.0 for Cu).[4]

Resonance Raman. Resonance Raman spectra were measured in solution state at –90°C (Bruker cryostat) using a laser with 407 nm excitation (Kr^+-laser) equipped with a Horiba Jobin-Yvon LabRAM HR800 confocal Raman spectrometer. The

concentrations of samples were between 1-2 mM. Spectra were obtained at a laser power of 2 mW with an accumulation time of about 10 min.

Attenuated total reflection Fourier-transform infrared spectroscopy (ATR-FTIR). IR spectra were measured at an AIGILENT Cary 630 FTIR spectrometer using a Diamond ATR sampling accessory with a type IIa diamond.

UV-vis spectroscopy. The UV–Vis absorption spectra were recorded with an 8453 UV-Visible Spectroscopy system from Agilent. The measurements were carried out in 10 mm precision cuvettes made of SUPRASIL® quartz glass, the closures of which were equipped with a septum. The measurements at low temperatures were carried out by cooling the cuvette holder using a cooling thermostat USP-203-A from Unisoku Scientific Instruments. The analysis of the spectra was carried out with the software UV–Visible Chemstation from Agilent.

EPR spectroscopy. CW EPR spectra were collected by using a Bruker EMXplus Instrument at a frequency of ca. 9.35 GHz (X-band). Samples were measured at an average temperature of 13 K as frozen solutions (powder spectra) by the use of a liquid helium recirculating cooling system provided by ColdEdge. X-band CW EPR spectra at liquid nitrogen temperature were measured on an ESR Miniscope MS5000 (Magnettech), equipped with a quartz finger dewar. Experimental parameters: microwave frequency 9.44-9.45 GHz, microwave power 1 mW, modulation amplitude 0.8 mT, temperature 77 K. Quantification of the concentration of the paramagnetic species against a copper(II) standard of known concentration was carried out by calculation of the respective double integrals upon polynomial baseline correction.

1.3. Synthesis of compounds and intermediates

1.3.1. Synthesis [Cu(MeAN)]BF_4, [Cu(AN)]BF_4 and [Cu(AN-D)]BF_4

[Cu(MeAN)]BF_4 and **[Cu(AN)]BF_4** were synthesized according to an adapted literature procedure.[5]
300 mg tetrakisacetonitrile copper tetrafluoroborate (0.95 mmol) were added to a solution of 2,6,10-trimethyl-2,6,10-triazaundecane (MeAN) (300 µL, 1.24 mmol, 1.3 eq) or 3,3′-Iminobis(N,N-dimethylpropylamine) (AN) (200 µL, 1.27 mmol, 1.3 eq) in 5 ml dry and degassed DCM. Both ligands were degassed using freeze-pump-thaw technique and dried for one hour over activated molecular sieve (4 Å) prior to use. The clear solutions that were received after stirring under inert conditions for one hour were added to 20 ml of dry, degassed hexane. In both cases off-white precipitates were formed. The solids were collected by decantation of the left-over solution. Afterwards the solids were washed three times with diethyl ether and dried under vacuum.

285 mg (0.79 mmol, 84%) of [Cu(MeAN)]BF_4 were received as an off-white solid.

ESI-MS (positive mode): $[Cu(MeAN)]^+$ calculated: 264.1, found 264.1

245 mg (0.73 mmol, 76%) of [Cu(AN)]BF_4 were received as a light yellow solid.

ESI-MS (positive mode): $[Cu(AN)]^+$ calculated: 250.1, found 250.0
ATR-FTIR: ν (cm^{-1}) = 520, 770, 898, 972, 1029, 1045, 1137, 1460, 2840, 3280 (N–H).

[Cu(AN–D)]BF_4 was prepared in a similar way like [Cu(AN)]BF_4 starting with deuterated ligand AN–D. For deuteration of the ligand, 100 µL AN were stirred for 24 h in 1 ml MeOD. The solvent was removed under vacuum and the procedure was once repeated to ensure complete deuteration. During the complexation d_2-DCM was used to prevent an exchange of the N-D with proton. [Cu(AN-D)]BF_4 (92 mg, 0.30 mmol, 64%) was received as a light yellow solid.

ESI-MS (positive mode): $[Cu(AN-D)]^+$ calculated: 251.1, found 251.1

ATR-FTIR: ν (cm^{-1}) = 520, 735, 770, 933, 972, 1029, 1045, 1132, 1460, 2431 (N-D), 2840.

1.3.2. Generation of intermediates 1, 2 and 3 and their ^{57}Fe- and ^{18}O-enriched variants

For generation of **3**, a 1-2 mM solution of [Fe(*trans*-cyclam)($CH_3CN)_2$](OTf)$_2$ in dry, degassed acetone was prepared, cooled to −60 °C and treated with O_2 by bubbling through a cannula into the cool solution for 10 s. After 1000 s intermediate **3** is formed in almost quantitative yield. The reaction can be monitored using UV-vis spectroscopy by observing the increase of the 330 nm band.[6]

In a typical experiment, intermediate **2** is generated by addition of a 0.1 ml of a 10 mM solution of [Cu(MeAN)]BF_4 in acetonitrile to 1 ml of a 1 mM degassed solution of **3** below –60 °C. Excess O_2 in the solution of **3** needs to be removed by bubbling of argon for 100 s prior to the addition of [Cu(MeAN)]BF_4.

Intermediate **1** is generated in a similar way like **2** but addition of [Cu(AN)]BF_4 to the solution of **3** needs to happen at –90°C. Removal of excess O_2 is not necessary in this case.

1.3.3. Generation of 1-$^{p\text{-X}}$PhO•, 1-DCHIm and 2-DCHIm

In a typical experiment a 1 mM solution of **1** was treated with 0.1 ml of a 100 mM solution of the substituted phenol in acetone at –90 °C. The formation of **1-$^{p\text{-X}}$PhO•** was followed by UV-vis spectroscopy.

1-DCHIm and **2**-DCHIm were generated by addition of 0.1 ml of a 20 mM solution of DCHIm in acetone to 1 ml of 1 mM solutions of **3**. Afterwards [Cu(MeAN)BF_4] and [Cu(AN)BF_4], were added as described above at –92.5 °C to yield **1**-DCHIm and **2**-DCHIm respectively.
Alternatively, 2 eq of DCHIm were added to solutions of **1** or **2** at –92.5 °C, but the life time of **1**-DCHIm and **2-**DCHIm would be reduced by half compared to the procedure described above.

1.4. Reactivity studies and determination of rate constants

Reactivity studies were usually performed by addition of given equivalents of the corresponding substrates that were dissolved in 0.1 ml of dry degassed acetone to solutions of the respective intermediates **1**, **2**, **3**, **1**-DCHIm and **2**-DCHIm via a syringe. The reaction was followed by UV-vis spectroscopy.

For reactivity studies using GC-MS analysis, the respective intermediates were first generated and typically 5 eq of substrates were added as described previously. After the reaction was complete 1 eq of biphenyl was added as an internal standard and the reaction mixture was transferred on a mini-column of silica and $MgSO_4$ x $2H_2O$ to get rid of the metal complex. Afterwards the sample was injected into the GC-MS with an autosampler as described previously.

Pseudo-first order rate constants k_{obs} were received by following and plotting the decay of the absorption maximum bands (615 nm for **1**). For reactions of intermediate **2** and **3**, the formation of the absorption features of [Fe^{IV}(O)(trans-cyclam)(CH_3CN)]$^{2+}$ and **2-Cu** were followed at 690 nm and 680 nm respectively. For determining the second order rate constant k_2 was received by plotting a linear fit for k_{obs} versus the initial substrate concentration. See

Figure S 20, Figure S 21, Figure S 28, Figure S 29 and Figure S 30.

2. Theoretical section

All calculations were carried out using ORCA 5.0.3.[7-9] Relativistic effects were considered using the zeroth-order regular approximation (ZORA).[10-12] Specially adapted segmented all-electron relativistically recontracted[13] basis sets were used, ZORA-TZVP for Fe, Cu, O and N atoms and ZORA-SVP for C and H atoms. The conductor-like polarizable continuum model (C-PCM)[14] with the solvent properties of acetone was used. Tight self-consistent field (TightSCF) convergence criteria were employed. The atom-pairwise dispersion correction with Becke-Johnson damping (D3BJ)[15] was applied to the DFT energy.

Geometry optimizations and frequency calculations were performed using the density functional BP86[16, 17] with the resolution of identity (RI) approximation together with decontracted auxiliary SARC/J Coulomb fitting basis sets. Geometry optimizations were performed in the high-spin (triplet) state for all models and additionally in the broken-symmetry (BS-)DFT (singlet) state for most of them, which lead to similar structures of slightly higher-energies throughout.

The hybrid functional B3LYP[17-19] with the RI approximation to the Coulomb exchange and the chain-of-spheres approximation to exact exchange (RIJCOSX)[20, 21] along with SARC/J auxiliary basis sets was employed in calculations of final DFT energies, EPR properties (zero-field splitting), as well as exchange couplings by the BS-DFT approach, and transitions to electronic excited states using (full) time-dependent (TD-)DFT. Exchange coupling constants J based on the isotropic Heisenberg-Dirac-van Vleck Hamiltonian $\hat{H} = -2J\hat{\boldsymbol{S}}_{\boldsymbol{1}}\hat{\boldsymbol{S}}_{\boldsymbol{2}}$ were extracted from the energies and spin expectation values of the high-spin and BS solutions following Yamaguchi's approach.[22]

Optimized structures, as well as difference densities and natural transition orbitals (NTOs)[23] were visualized in PyMol[24] and UCSF Chimera.[25]

3. Supplementary Tables and Figures

3.1. Supplementary Figures S1-34

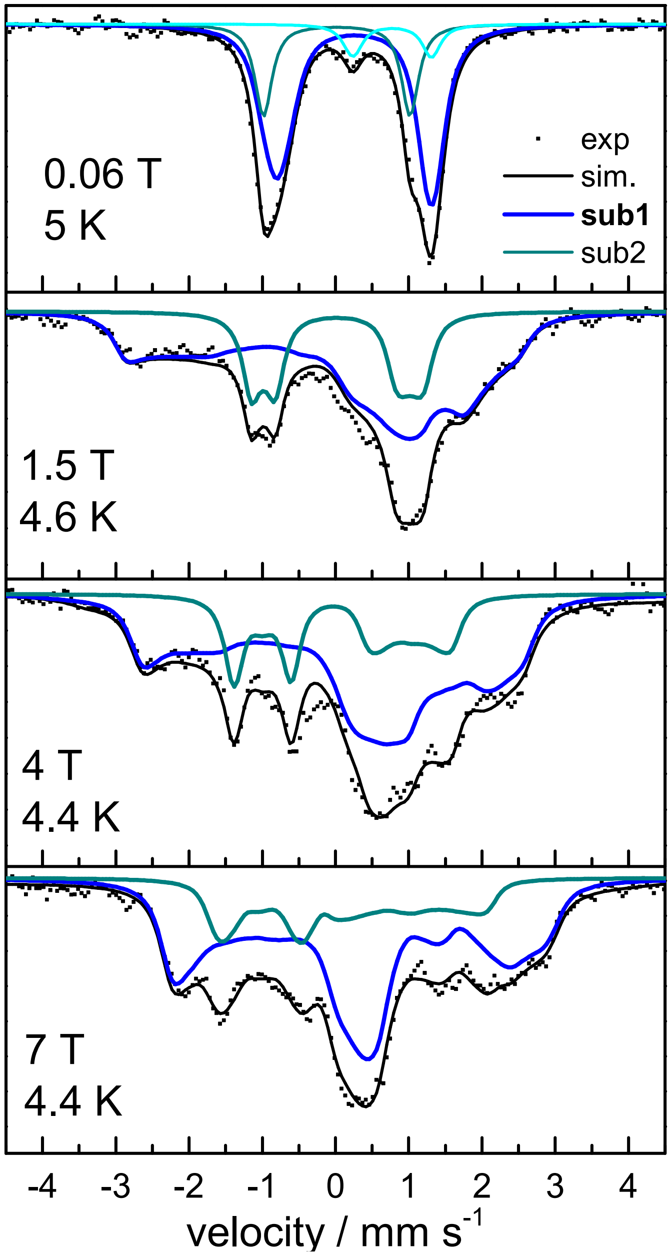

	Sub1	Sub2	Sub3
%	70	24	6
S_t	1	1	
g	2.0 (iso)	2.0 (iso)	
D_t	1.5 (±0.5) cm^{-1}	16.1 (±1.0) cm^{-1}	
E/D	0.170 (±0.1)	0.187 (±0.1)	
δ	0.255 mm s^{-1}	0.017 mm s^{-1}	0.77 mm s^{-1}
ΔE_Q	-2.109 mm s^{-1}	2.001 mm s^{-1}	1.07 mm s^{-1}
η	0.432	0.002	
Γ	0.35 mm/s	0.27 mm/s	
A_{xx}, A_{yy}, A_{zz}	(-3.2, -16.2, 4.4) T	(-20.3, -13.0, -5.7) T	

*Figure S 1 Mößbauer spectra under applied magnetic field of ^{57}Fe-enriched **1** (1 mM) in frozen solution of acetone/CH₃CN 10/1 v/v (a small high-spin Fe^{II} impurity visible only at low field spectra (cyan) was not simulated in the high field spectra).*

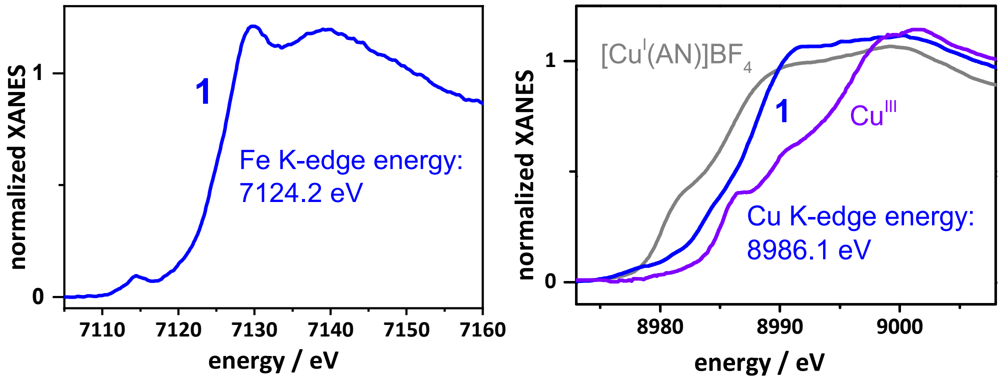

*Figure S 2 XANES spectra at the Fe K-edge (left) and at the Cu K-edge (right) of **1** (blue lines), compared to spectra of monovalent copper in [Cu^{I}(AN)]BF_4 (grey line) and of trivalent copper in $KCu^{III}O_2$ (purple line, spectrum reproduced from ref[26]) as reference materials.*

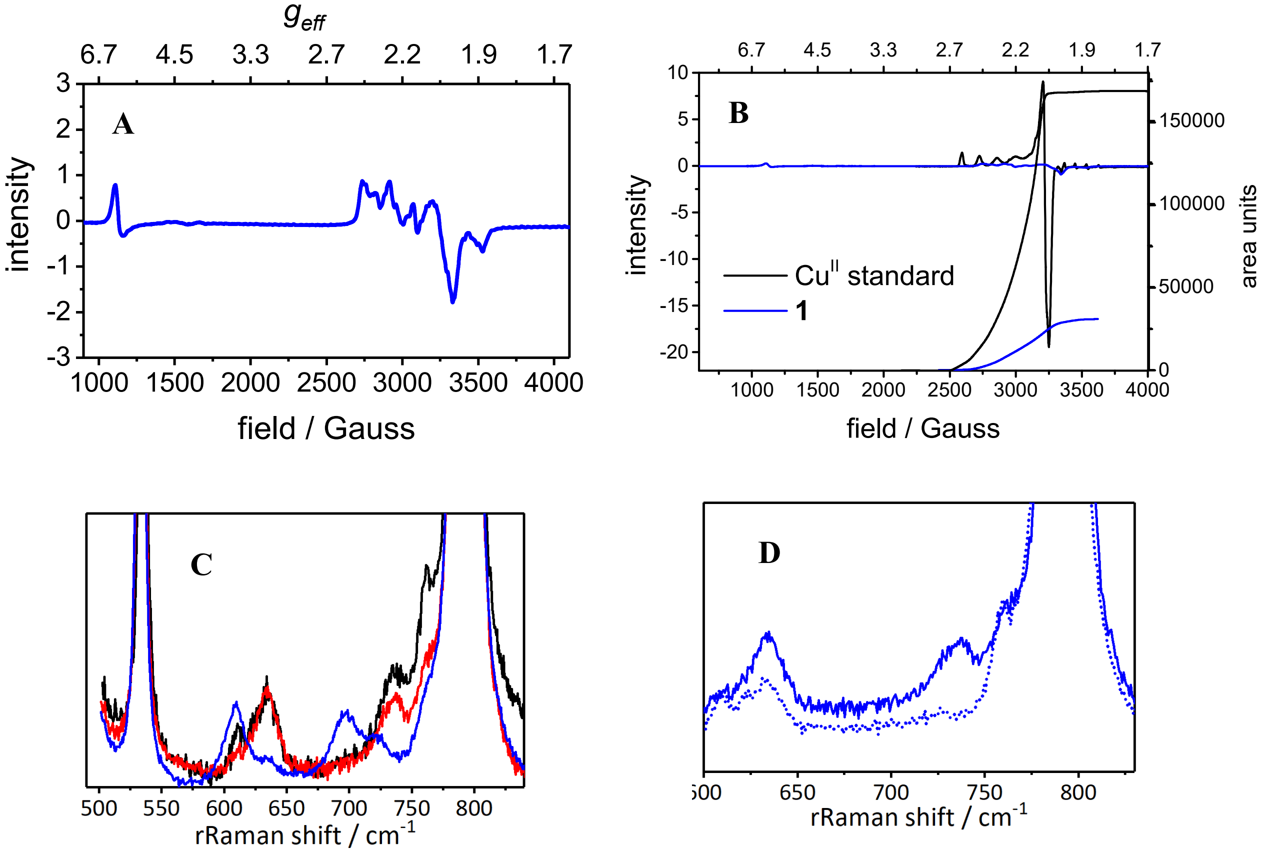

*Figure S 3. A) X-band EPR spectrum of a 1 mM solution of **1** in acetone/CH_3CN 10/1 v/v and B) Comparison of the EPR intensity to a 1.2 mM Cu^{II} standard ($CuSO_4$ in MeOH) measured under the same conditions (microwave frequency 9.637 GHz, microwave power 0.06 mW, modulation amplitude 4.996 G, temperature 13 K) (area units after double integration: 26 000 for **1** and 164 000 for the Cu^{II} standard); C) RRaman spectra of two different samples of **1** (red and black), and ^{18}O-labelled **1** (blue) measured in a 10/1 acetone/CH_3CN v/v solution (407 nm excitation, 2 mW, –90 °C); D) RRaman spectra of **1** (bold line) and its thermal decay product (dashed line) measured in a 10/1 acetone/CH_3CN v/v solution (407 nm excitation, 2 mW, –90 °C)*

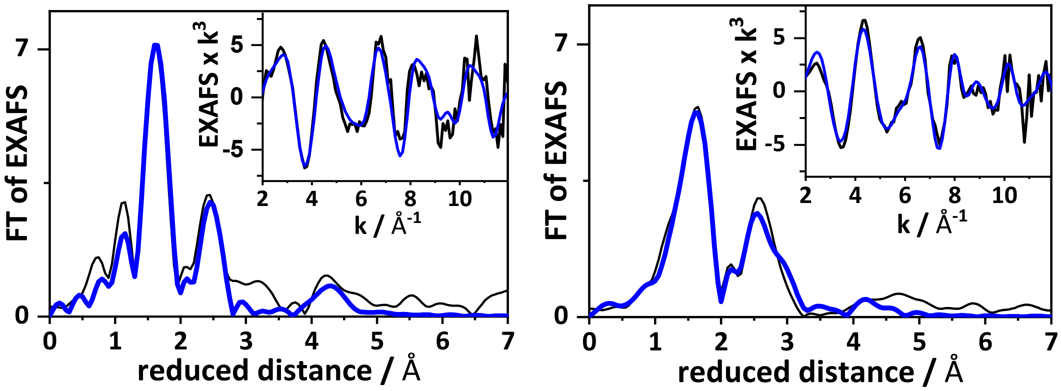

Figure S 4 Fourier-transforms of EXAFS spectra at the Fe K-edge (left) and the Cu K-edge (right) of ***1*** *and the respective* k^3*-weighted EXAFS spectra in the insets (experimental data, black lines; simulations, blue lines, parameters in Tables S3 and S4).*

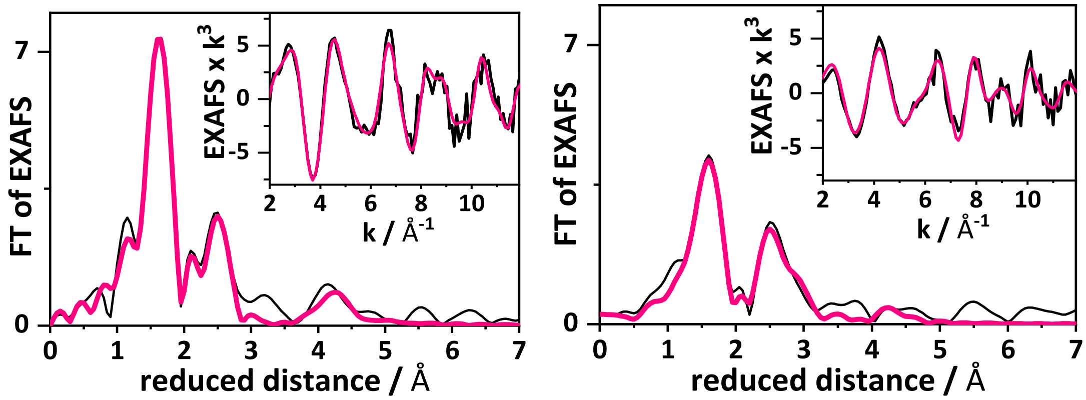

Figure S 5 Fourier-transforms of EXAFS spectra at the Fe K-edge (left) and the Cu K-edge (right) of ***2*** *and the respective* k^3*-weighted EXAFS spectra in the insets (experimental data, black lines; simulations, pink lines, parameters in Tables S3 and S4).*

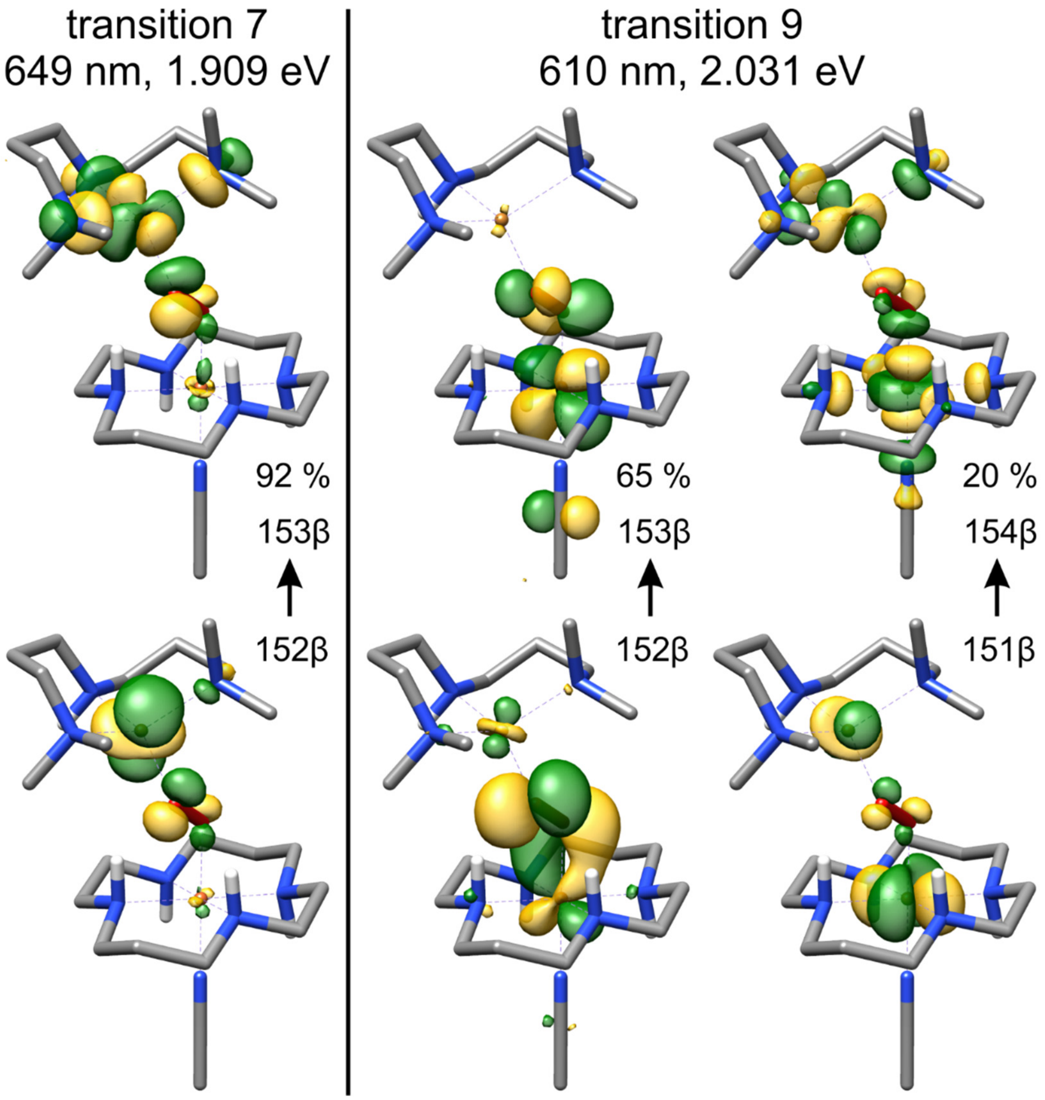

*Figure S 6 TD-DFT-based NTOs (isovalue 0.04 a.u.) for the charge-transfer excitations in **1-trans-b** from the electronic ground state into excited states 7 and 9, giving rise to the intense band peaking at 617 nm (Figure 3B).*

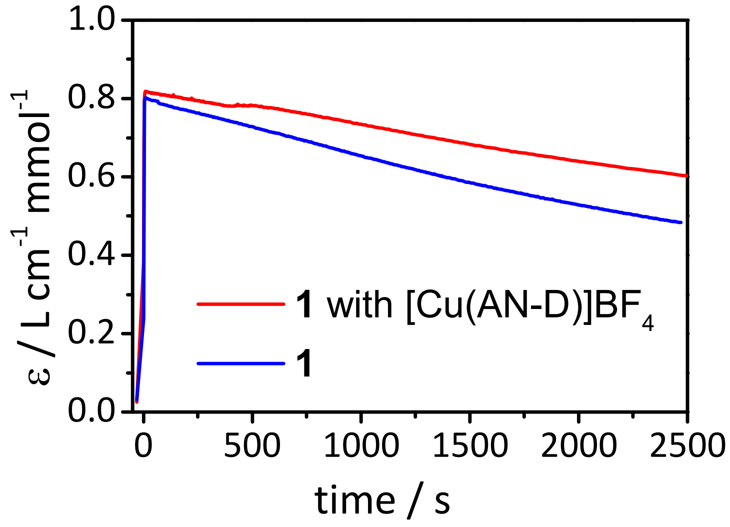

*Figure S 7 Comparison of the self-decay of **1** (blue) and **1** prepared with $[Cu^I(AN\text{-}D)]BF_4$ (red) by tracking the 615 nm absorption feature at −90 °C.*

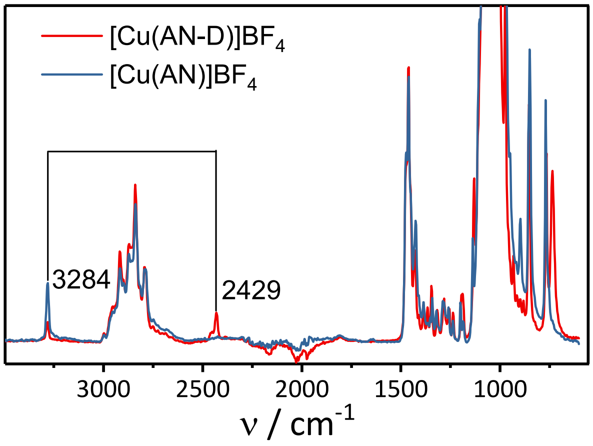

Figure S 8 ATR-IR spectra of [Cu(AN)]BF$_4$ (blue) and [Cu(AN–D)]BF$_4$ (red) showing the shift of the N–H-vibration at 3284 cm^{-1} upon deuteration of the ligand.

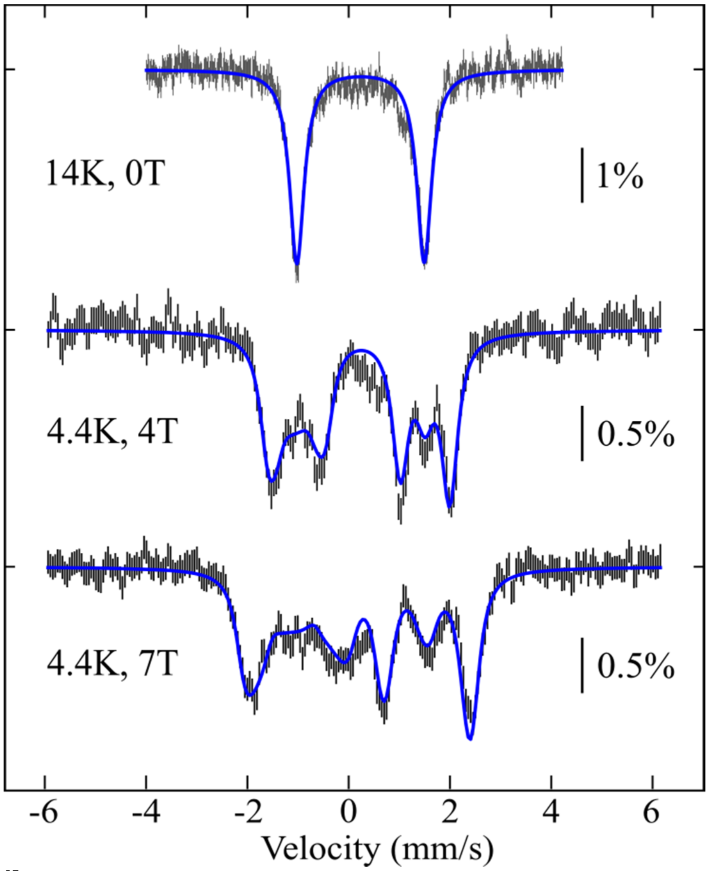

*Figure S 9 Mößbauer spectra of ^{57}Fe-enriched **2** under applied magnetic field and different temperatures. The spectra were simulated with a single species with $S_t = 0$, $\delta = 0.235$ mm s^{-1}, $\Delta E_Q = -2.514$ mm s^{-1}, $\eta = 0.398$ and $\Gamma = 0.23$ mm s^{-1} (experimental data grey).*

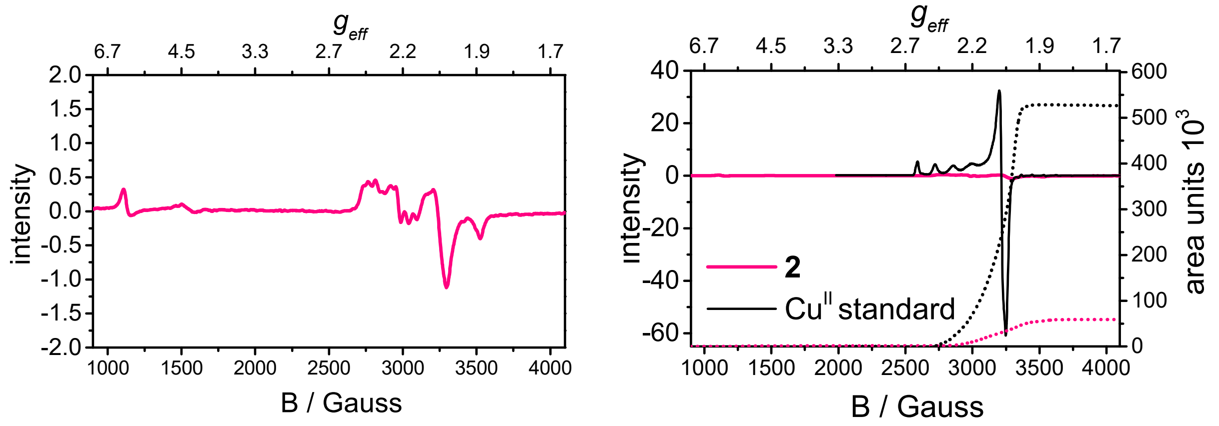

*Figure S 10 X-band EPR spectrum of a 1 mM solution of **2** in acetone/CH_3CN 10/1 v/v (left) and comparison to a 1.2 mM Cu^{II} standard ($CuSO_4$ in MeOH) measured under the same conditions for comparison (right) (microwave frequency 9.353 GHz, microwave power 1 mW, modulation amplitude 5.000 G, temperature 12 K) (area units after double integration: 59 000 for **2** and 530 000 for the Cu^{II} standard)*

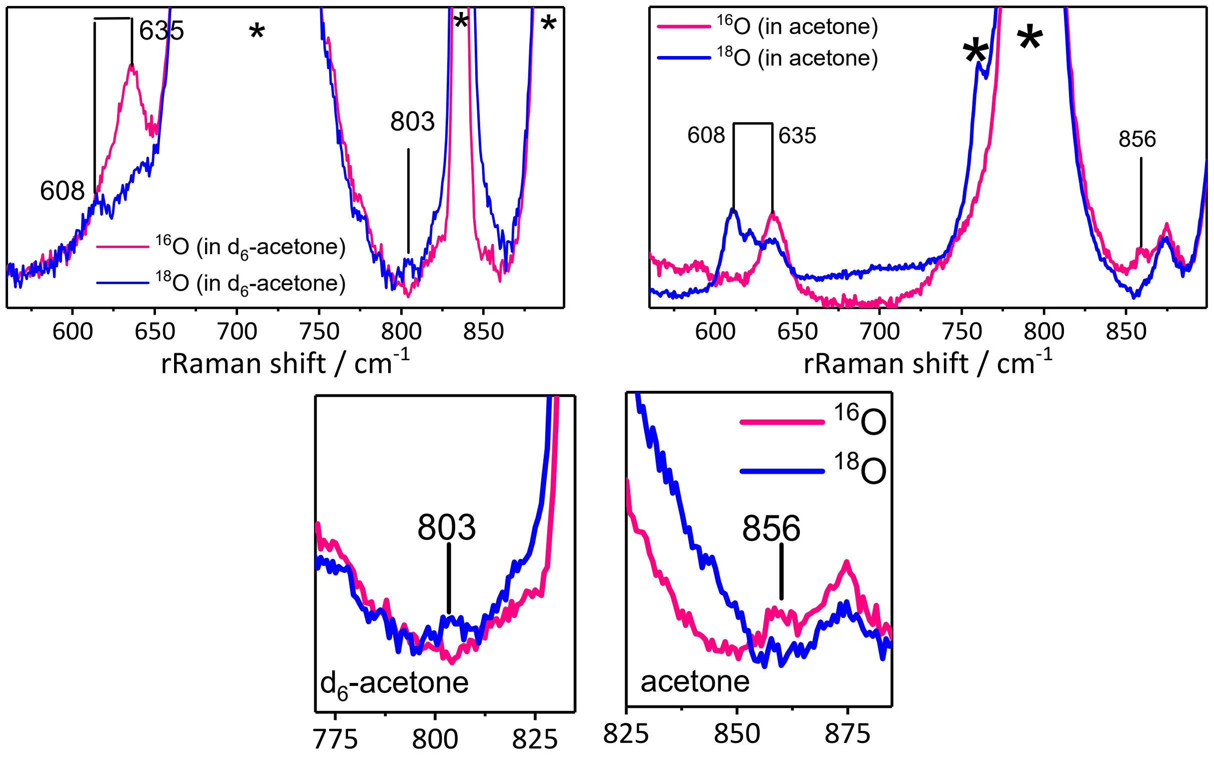

*Figure S 11 rRaman spectra of **2** (pink) and ^{18}O-enriched **2** (blue) in a d_6-acetone/CD_3CN 10/1 v/v mixture (left) and in an acetone/CH_3CN 10/1 v/v mixture (right) (–90 °C, 2 mW, 407 nm excitation). Below a cut out of both spectra for a better identification of the ~800 cm^{-1} feature. Solvent features are marked by asterisks.*

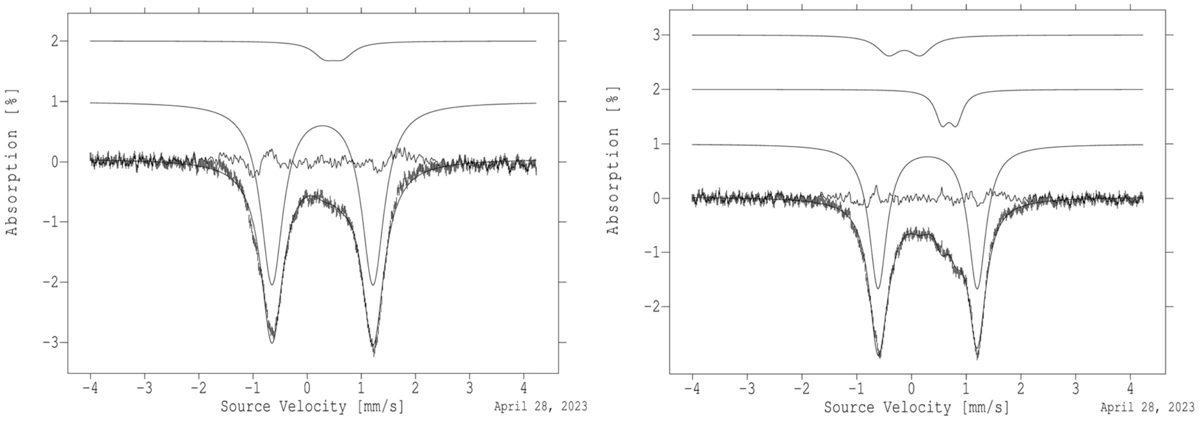

*Figure S 12 Mößbauer spectrum of ^{57}Fe-enriched **1**-DCHIm (sub1: δ = 0.28 mm s^{-1}, $|\Delta E_Q|$ = 1.87 mm s^{-1}, 92%; sub2: δ = 0.48 mm s^{-1}, $|\Delta E_Q|$ = 0.33 mm s^{-1}, 8%) (left) and of ^{57}Fe-enriched **2**-DCHIm (sub1: δ = 0.29 mm s^{-1}, $|\Delta E_Q|$ = 1.81 mm s^{-1}, 79%; sub2: δ = 0.69 mm s^{-1}, $|\Delta E_Q|$ = 0.26 mm s^{-1}, 11%; sub3: δ =0.10 mm s^{-1}, $|\Delta E_Q|$ = 0.58 mm s^{-1}, 10%)*

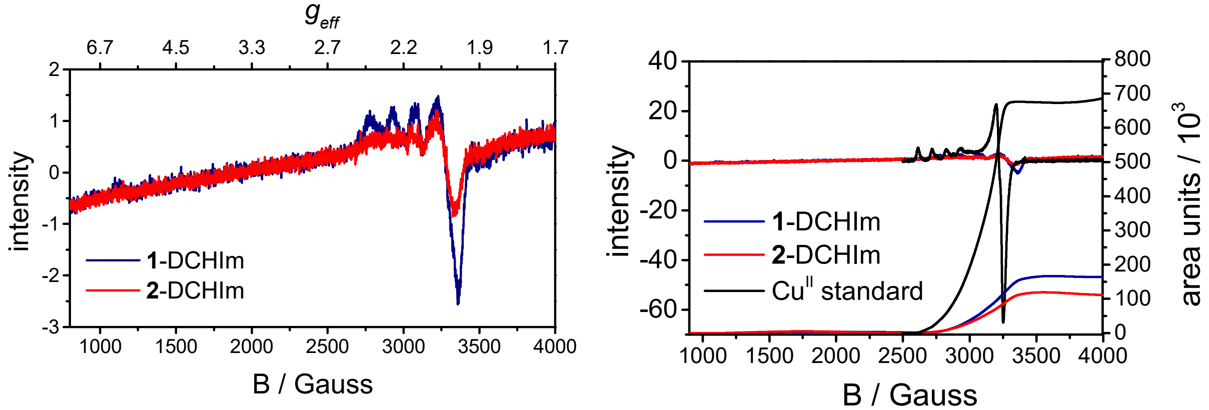

*Figure S 13 X-band EPR spectrum of 1 mM solutions of **1**-DCHIm and **2**-DCHIm in acetone/CH_3CN 10/1 v/v (left) and comparison to a 1.2 mM Cu^{II} standard ($CuSO_4$ in MeOH) measured under the same conditions for comparison (right) (9.44-9.45 GHz, microwave power 1 mW, modulation amplitude 0.8 mT, temperature 77 K) (area units after double integration: 166 000 for **1**-DCHIm and 120 000 for **2**-DCHIm 675 000 for the Cu^{II} standard)*

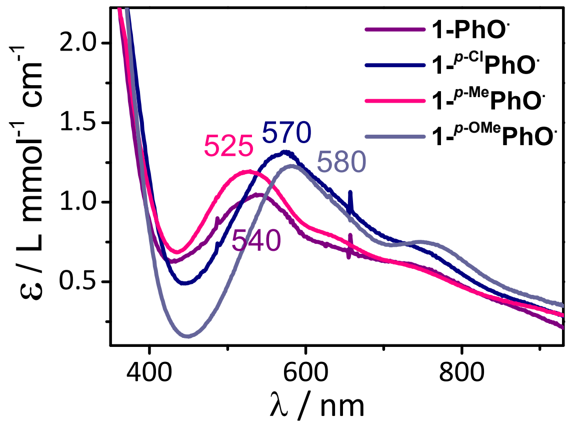

*Figure S 14 UV-vis spectra after the conversion of **1** into **1-PhO•** (purple), **1-$^{p\text{-}Cl}$PhO•** (dark blue), **1-$^{p\text{-}Me}$PhO•** (pink) and **1-$^{p\text{-}OMe}$PhO•** (grey) upon addition of 5 eq of phenol, 4-chlorophenol, p-cresol and 4-methoxyphenol respectively (acetone/CH_3CN 10/1, –90°C).*

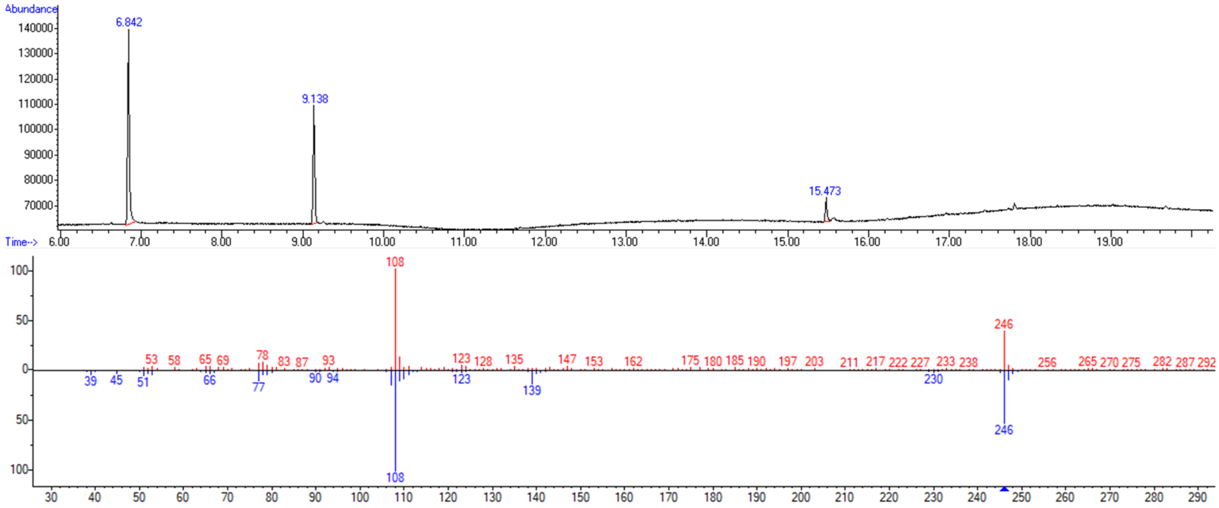

*Figure S 15 GC FID spectrum for the reaction of **1** with ${}^{p\text{-}OMe}$PhOH (after warm-up and decay of **1-${}^{p\text{-}OMe}$PhO•**) showing the formation of 5,5'-Dimethoxy[1,1'-biphenyl]-2,2'-diol (15.473 min) in approx. 20% yield (determined by comparison to an internal biphenyl (9.318 min) standard and an estimated calibration constant k of around 1 (based on the amount of C-atoms, that determine the FID-response and comparison to the k values of other dihydroxybiphenyls)) (top) and the mass spectrum at 15.473 min (red) compared to the bibliographical reported mass spectrum of 5,5'-Dimethoxy[1,1'-biphenyl]-2,2'-diol (blue) (bottom).*

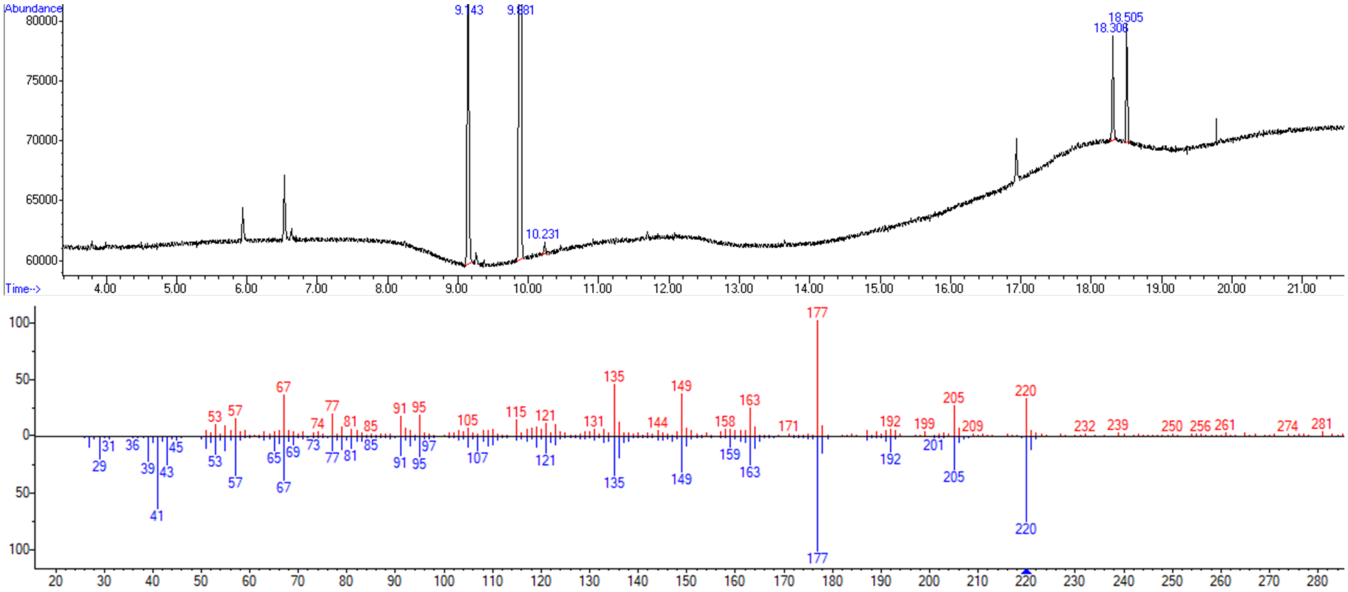

*Figure S 16 GC FID spectrum for the reaction of **1** with 2,6-ditertputylphenol (2,6-dtbp) (after warm-up) showing the formation of 2,6-di-tert-butylcyclohexa-2,5-diene-1,4-dione (diketone, 10.235 min, 18%), 3,3',5,5'-tetra-tert-butyl-[1,1'-biphenyl]-4,4'-diol (dimer-OH, 18.307 minutes, 13%) and 3,3',5,5'-tetra-tert-butyl-[1,1'-bi(cyclohexylidene)]-2,2',5,5'-tetraene-4,4'-dione (dimer-ketone 18.505 min, 135%) (determined by comparison to an internal biphenyl standard (9.318 min) and a previously determined calibration constant) (top) and the mass spectrum at 10.235 min (red) compared to the bibliographical reported mass spectrum of 2,6-di-tert-butylcyclohexa-2,5-diene-1,4-dione (diketone, blue) (bottom).*

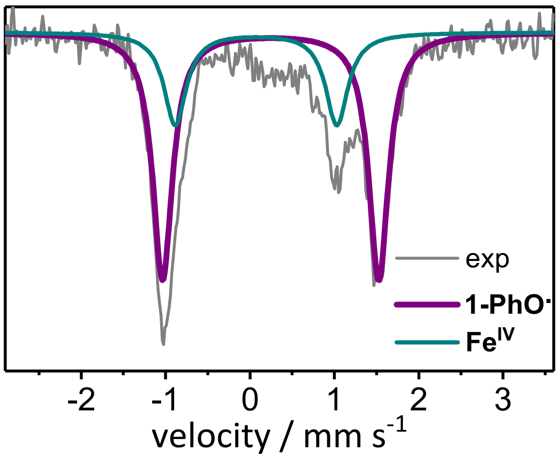

*Figure S 17 Mößbauer spectrum of ^{57}Fe-enriched **1-PhO•**, showing a main Fe^{III} low spin species (δ = 0.25 mm·s^{-1}, |ΔE_Q| = 2.57 mm·s^{-1}, 72%, Table S6), a Fe^{IV} species (δ = 0.07 mm·s^{-1}, |ΔE_Q| = 1.92 mm·s^{-1}, 28%) and an undefined Fe^{III} high -spin impurity (δ = 0.63 mm s^{-1}, |ΔE_Q| = 0.71 mm·s^{-1}, 14%).*

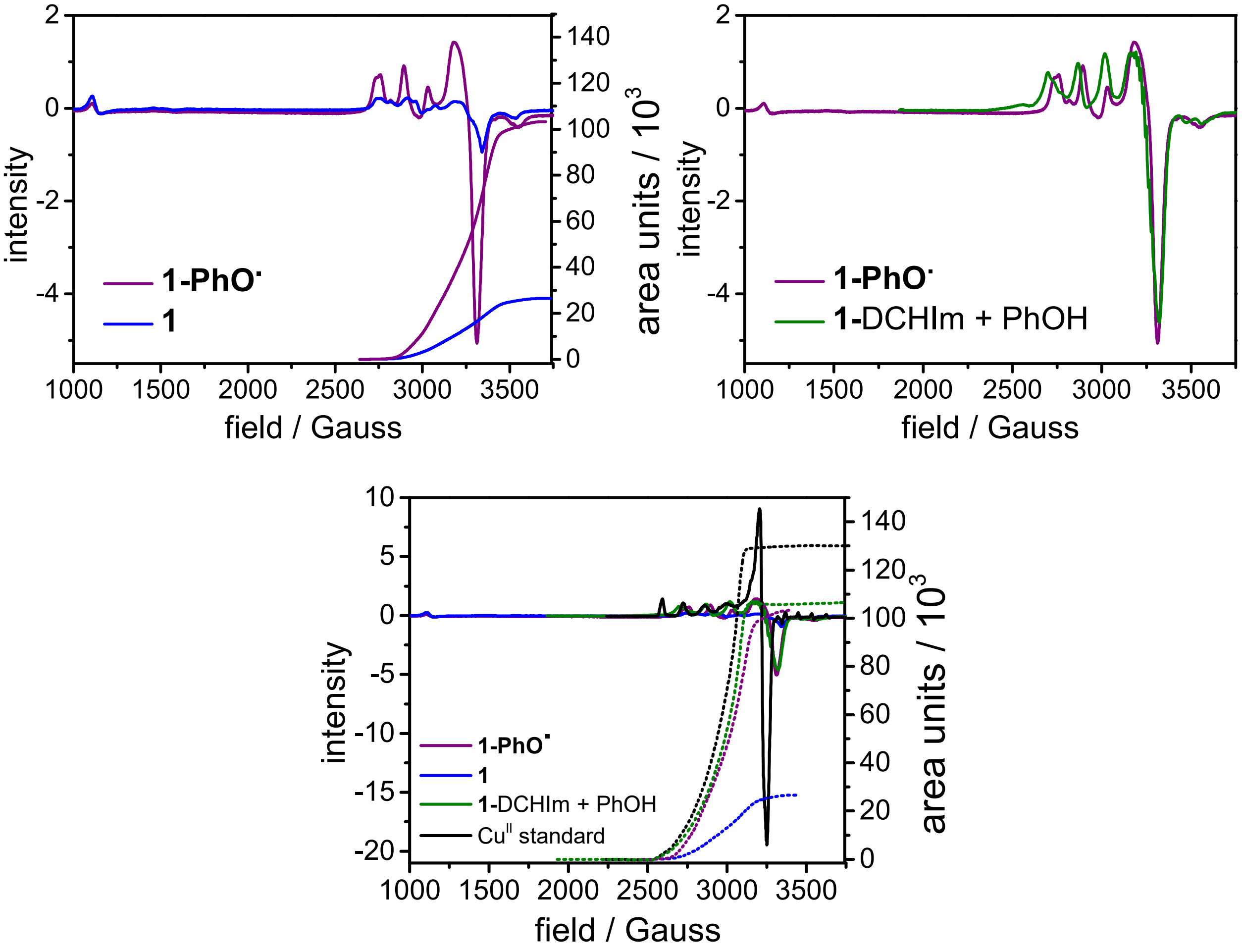

*Figure S 18 Top left: X-band EPR spectra of 1 mM solutions of **1-PhO•** and **1** in acetone/CH_3CN 10/1 v/v and comparison with the respective double integration curves (dotted lines) (microwave frequency 9.637 GHz, microwave power 0.06 mW, modulation amplitude 4.996 G, temperature 13 K) (area units after double integration: 26 000 for **1** and 103 000 for **1-PhO•**). Top right: X-band EPR spectra of 1 mM solutions of **1-PhO•** and **1**-DCHIm after reaction with 5 eq of phenol in acetone/CH_3CN 10/1 v/v. Bottom: X-band EPR spectra of 1 mM solutions of **1**, **1-PhO•** and **1**-DCHIm after reaction with 5 eq of phenol in acetone/CH_3CN 10/1 v/v mixtures and comparison with the a 1.2 mM Cu^{II} standard ($CuSO_4$ in MeOH) and the respective curves derived from double integration (dotted lines)(microwave frequency 9.637 GHz, microwave power 0.06 mW, modulation amplitude 4.996 G, temperature 13 K) (area units after double integration: 106 000 for **1**-DCHIm after reaction with 5 eq of phenol and 130 000 for the Cu^{II} standard)*

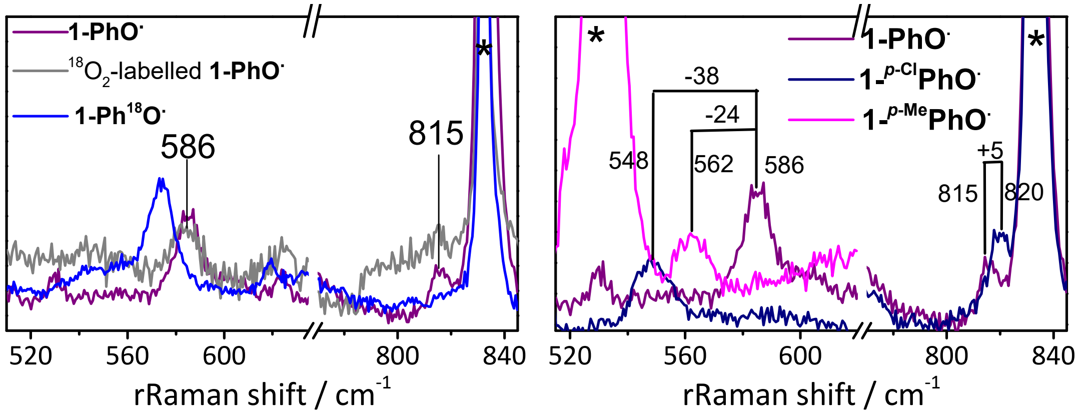

*Figure S 19 rRaman spectra of **1-PhO•** (purple), **1-PhO•** that was generated using $^{18}O_2$ (grey) and of **1-Ph^{18}O•** (blue) (left) and of **1-PhO•** (purple), **1-$^{p\text{-}Cl}$PhO•** (dark blue) and **1-$^{p\text{-}Me}$PhO•** (pink) (right) measured with 568 nm excitation (2 mW) in a d_6-acetone/CD_3CN 10/1 solution (**1-$^{p\text{-}Me}$PhO•** in acetone/CH_3CN 10/1) at –90 °C. Solvent features are marked by asterisks.*

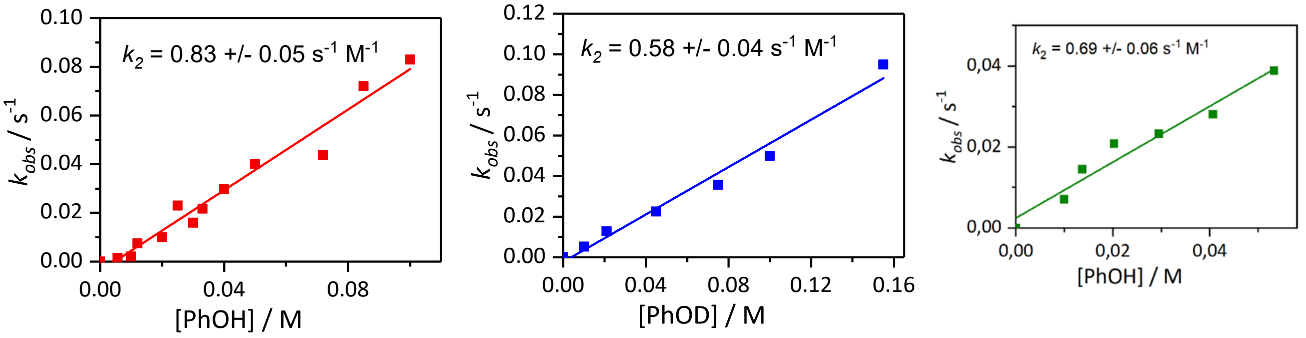

*Figure S 20 Pseudo first order plot for k_2 of the reaction of **1** with phenol (left) and phenol-D (middle) and **1** prepared with [Cu^I(AN-D)]BF_4 with phenol(right) forming **1-PhO•** .*

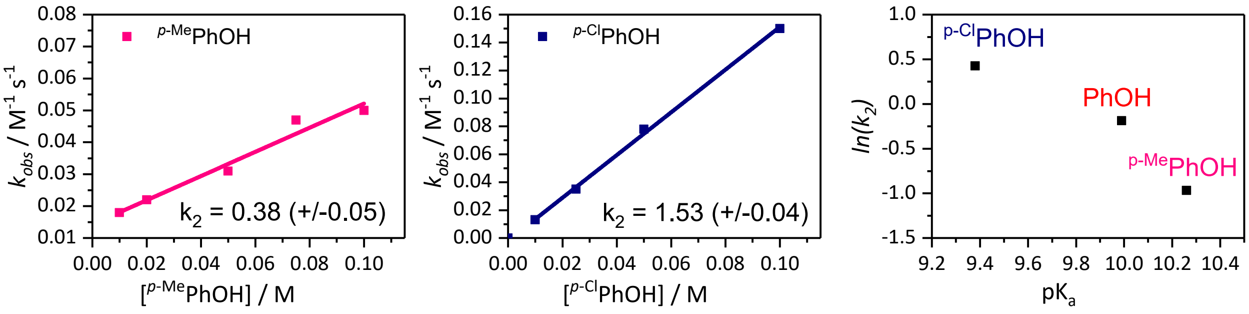

*Figure S 21 Pseudo first order plot for k_2 of the reaction of **1** with p-kresol (left) and p-chlorophenol (middle) forming **1-$^{p\text{-}Me}$PhO•** and **1-$^{p\text{-}Cl}$PhO•** respectively and the comparison of the logarithm of k_2 for different phenols against their pK_a (right).*

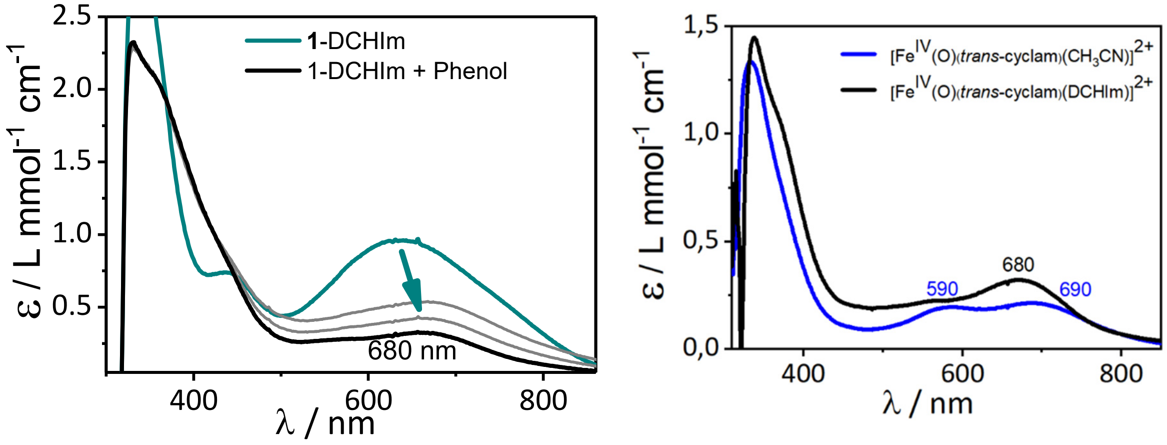

*Figure S 22 UV-vis spectra of the reaction of **1**-DCHIm with phenol (acetone/CH_3CN 10/1, –90 °C) (left) and of independently generated [Fe^{IV}(O)(trans-cyclam)(DCHIm)]$(OTf)_2$ (acetone/CH_3CN 10/1, –50 °C) (right) obtained by reaction of [$(CH_3CN)_2Fe^{II}$(trans-cyclam)]$(OTf)_2$ with oxygen at -50 °C followed by addition of DCHIm (5 equivalents). The UV-Vis absorption spectrum of [Fe^{IV}(O)(trans-cyclam)(CH_3CN)]$(OTf)_2$ is also shown for comparison purpose*

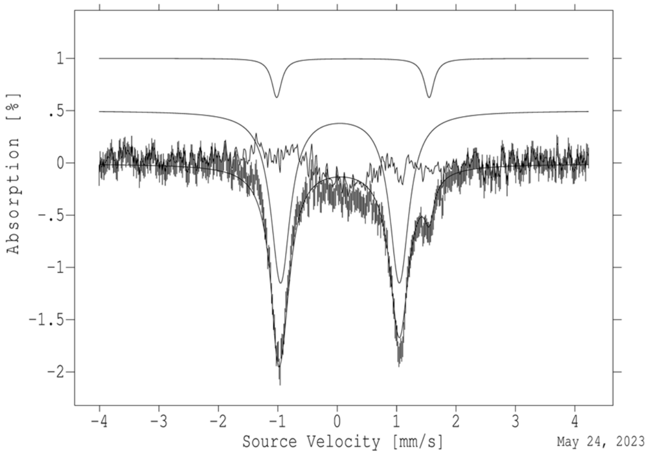

*Figure S 23 ^{57}Fe-Mößbauer spectrum of the reaction products of **1**-DCHIm with phenol (sub1: δ = 0.05 mm s^{-1}, $|\Delta E_Q|$ = 2.00 mm s^{-1}, 87%, sub2: δ = 0.27 mm s^{-1}, $|\Delta E_Q|$ = 2.56 mm s^{-1}, 13%).*

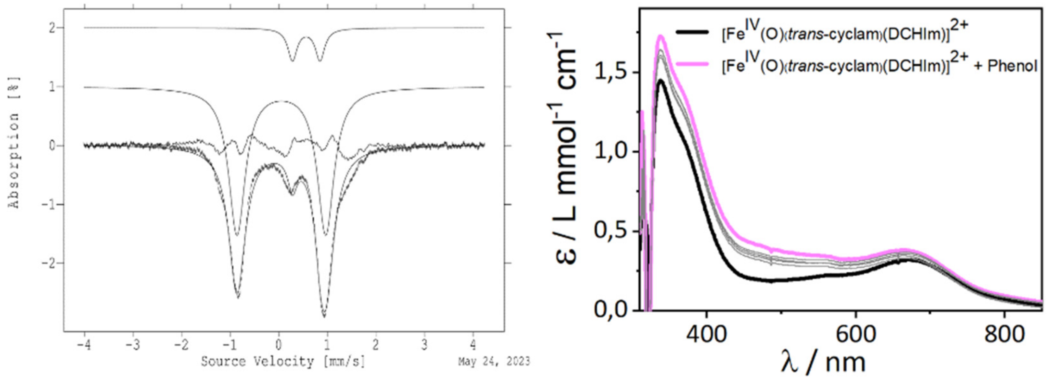

Figure S 24 Left: ^{57}Fe-Mößbauer spectrum of genuine [Fe^{IV}(O)(trans-cyclam)(DCHIm)]$(OTf)_2$ generated independently by reaction of [$(CH_3CN)_2Fe^{II}$(trans-cyclam)]$(OTf)_2$ with oxygen at -50 °C followed by addition of DCHIm (5 equivalents) (sub1: δ = 0.05 mm s^{-1}, $|\Delta E_Q|$ = 1.90 mm s^{-1}, 92%, sub2: δ = 0.57 mm s^{-1}, $|\Delta E_Q|$ = 0.56 mm s^{-1}, 8%). Right: Stability of the [Fe^{IV}(O)(trans-cyclam)(DCHIm)]$(OTf)_2$ core in presence of PhOH as evidenced from the retention of the characteristic near-IR feature at 680 cm^{-1} upon addition of PhOH to [Fe^{IV}(O)(trans-cyclam)(DCHIm)]$(OTf)_2$ at -50 °C.

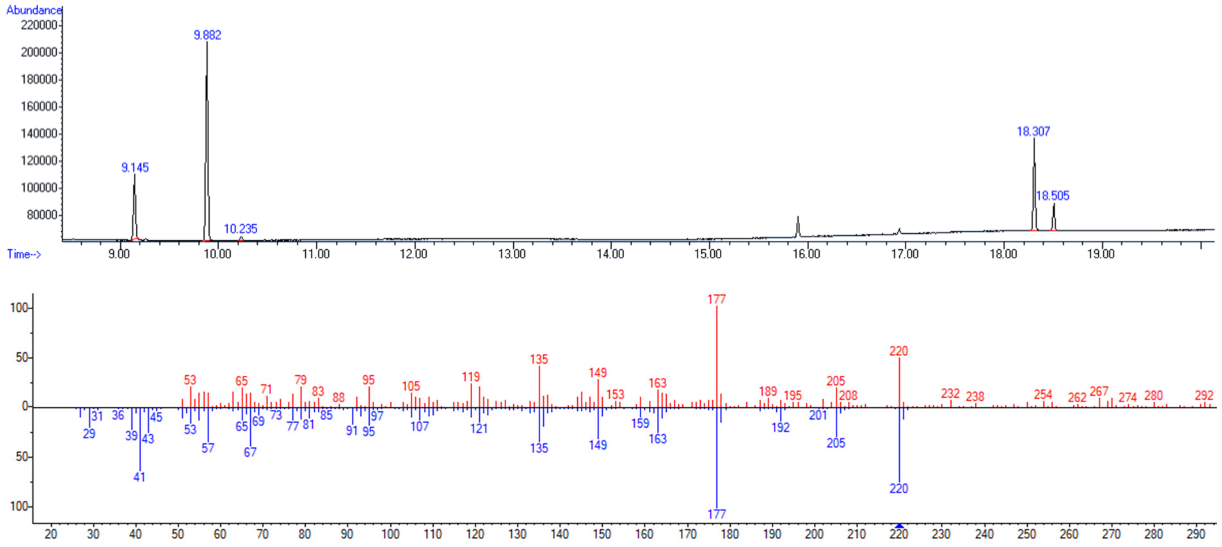

*Figure S 25 GC FID spectrum for the reaction of **2**-DCHIm with 2,6-ditertputylphenol (2,6-dtbp) (after warm-up) showing the formation of 2,6-di-tert-butylcyclohexa-2,5-diene-1,4-dione (diketone, 10.235 min, 18%), 3,3',5,5'-tetra-tert-butyl-[1,1'-biphenyl]-4,4'-diol (dimer-OH, 18.307 minutes, 13%) and 3,3',5,5'-tetra-tert-butyl-[1,1'-bi(cyclohexylidene)]-2,2',5,5'-tetraene-4,4'-dione (dimer-ketone 18.505 min, 135%) (determined by comparison to an internal biphenyl standard (9.318 min) and a previously determined calibration constant) (top) and the mass spectrum at 10.235 min (red) compared to the bibliographical reported mass spectrum of 2,6-di-tert-butylcyclohexa-2,5-diene-1,4-dione (diketone, blue) (bottom).*

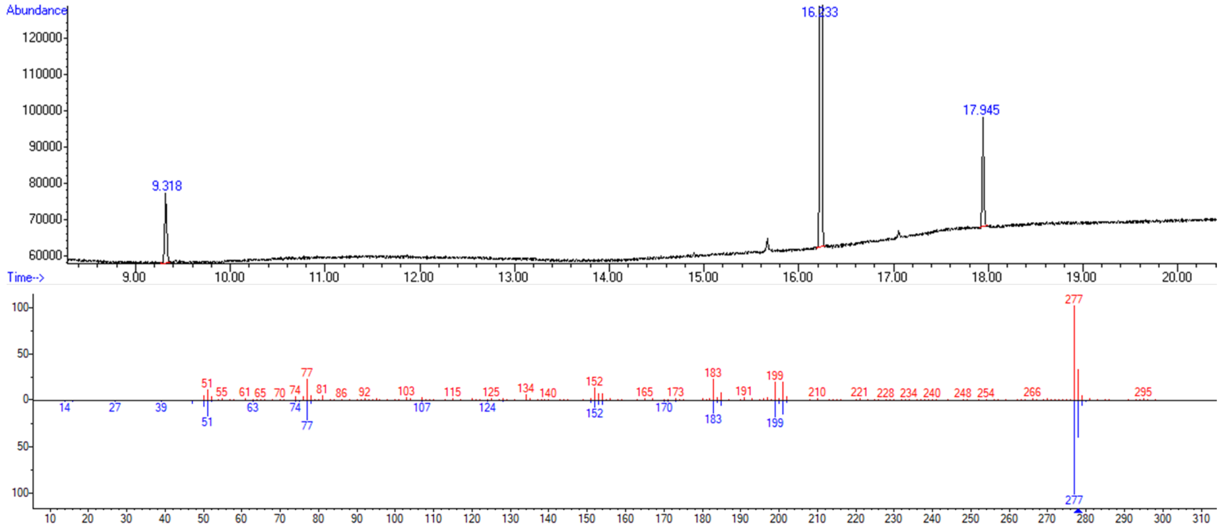

*Figure S 26 GC FID spectrum for the reaction of **2** with PPh_3 showing the formation of $OPPh_3$ (17.945 min) in 58% yield (determined by comparison to an internal biphenyl (9.318 min) standard) (top) and the mass spectrum at 17.945 min compared to the bibliographical reported mass spectrum of $OPPh_3$ (bottom).*

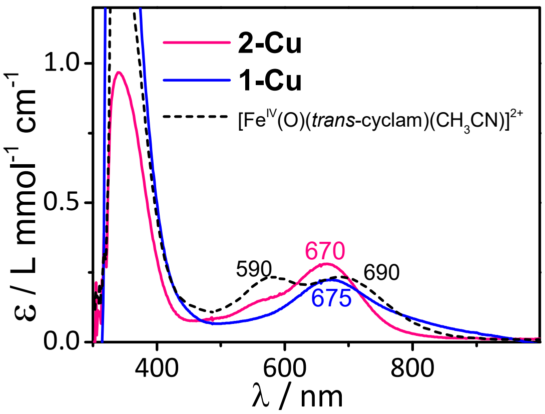

*Figure S 27 UV-vis spectra (acetone/CH_3CN 10/1) of **1** and **2** after reactions with 10 eq PPh_3 at –90 °C forming **1-Cu** and **2-Cu**, respectively. The spectra of the $[Fe^{IV}(O)(trans\text{-}cyclam)(CH_3CN)]^{2+}$ complex is also added for comparison.*

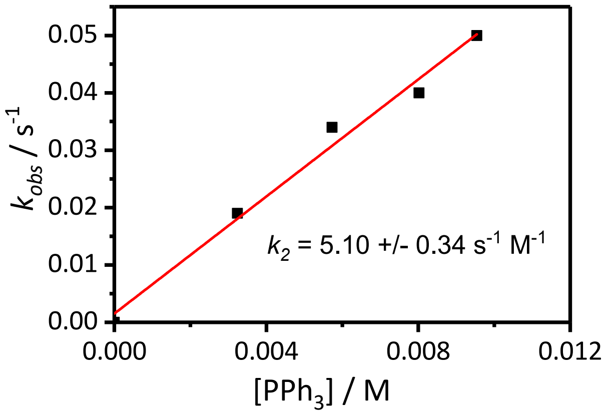

*Figure S 28 Plot of k_2 for the reaction of **1** with PPh_3 at –90 °C (fits for k_{obs} received by first order plots to the decrease of the 615 nm absorption feature).*

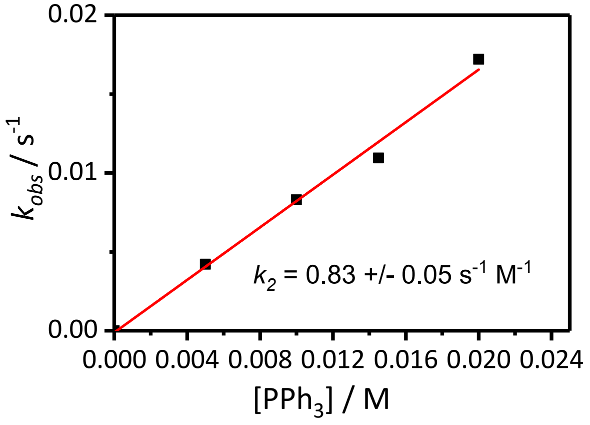

*Figure S 29 Plot of k_2 for the reaction of **2** with PPh_3 at –90 °C (fits for k_{obs} received by first order plots to the increase of the 680 nm absorption feature).*

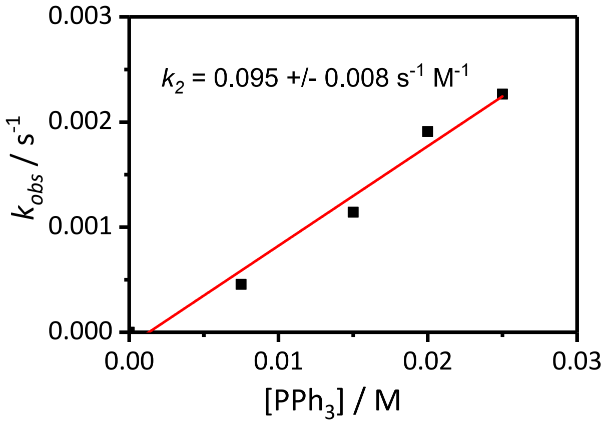

*Figure S 30 Plot of k_2 for the reaction of **3** with PPh_3 at –90 °C (fits for k_{obs} received by first order plots to the increase of the 690 nm absorption feature).*

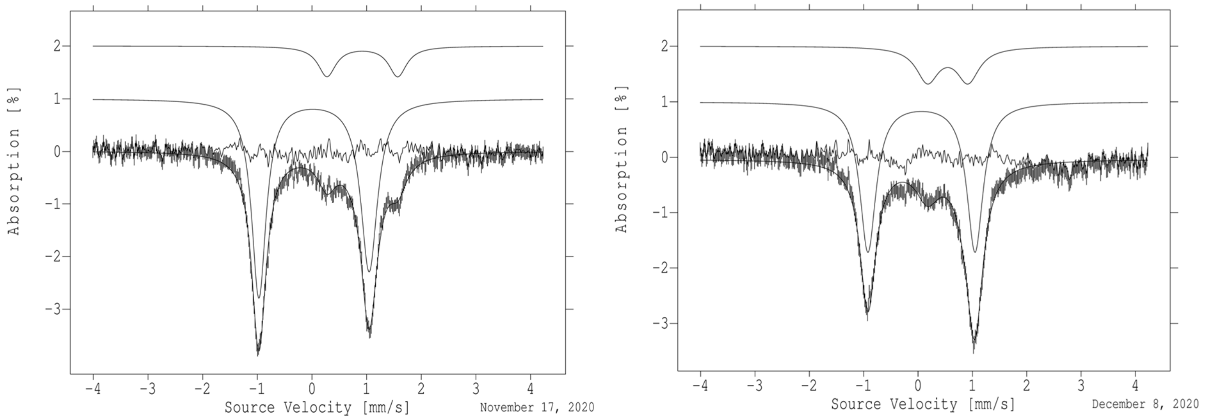

*Figure S 31 Mößbauer spectrum of ^{57}Fe-enriched **1-Cu** showing a main Fe^{IV} intermediate-spin species (δ = 0.04 mm s^{-1}, $|\Delta E_Q|$ = 2.01 mm s^{-1}, 86%) and an undefined Fe^{III} high-spin impurity (δ = 0.92 mm s^{-1}, $|\Delta E_Q|$ = 1.29 mm s^{-1}, 14%) (left) and of ^{57}Fe-enriched **2-Cu** (sub1: δ = 0.06 mm s^{-1}, $|\Delta E_Q|$ = 1.97 mm s^{-1}, 76%; sub2: δ = 0.68 mm s^{-1}, $|\Delta E_Q|$ = 0.72 mm s^{-1}, 24%) (right).*

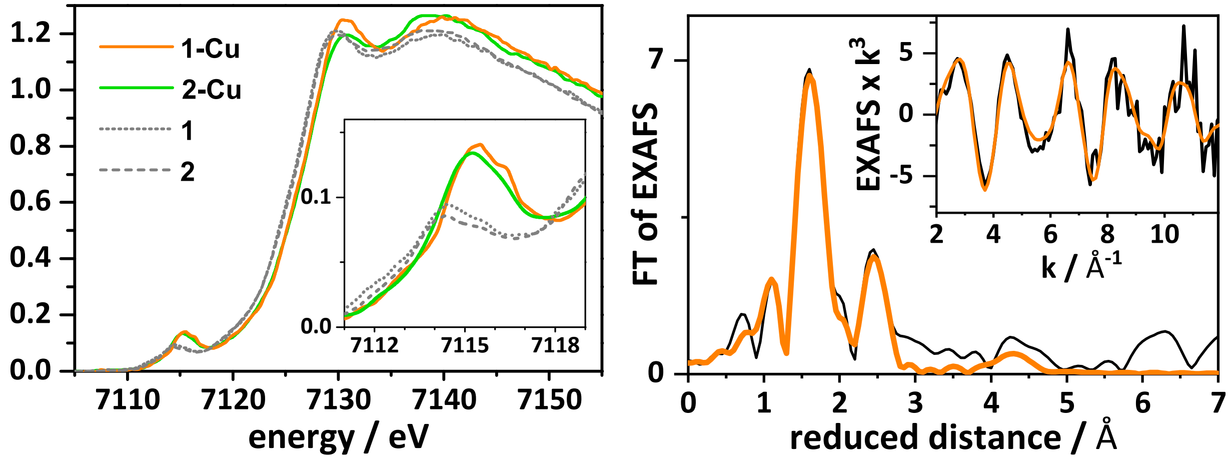

*Figure S 32 Left panel: XANES at the Fe K-edge of **1-Cu** (orange line, edge energy 7124.6 eV) and **2-Cu** (green line, edge energy 7124.7 eV) compared to spectra of **1** (grey dotted line) and **2** (grey dashed line). Inset: respective pre-edge features around 7115 eV in magnification. Right panel: Fourier-transform of EXAFS spectrum at the Fe K-edge of **1-Cu** and the respective k^3-weighted EXAFS spectrum in the inset (experimental data, black line; simulation, orange line, parameters in Table S3).*

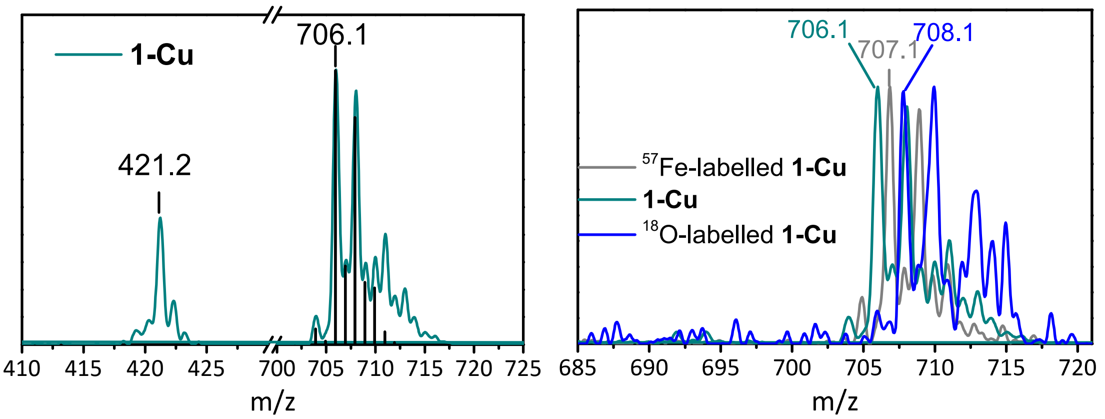

*Figure S 33 ESI-MS spectrum after decay of **1** (forming **1-Cu**) with the characteristic fragments at m/z = 421.2 and m/z = 706.1 assigned as [Fe(O)(cyclam)(OTf)Cu(AN)Cl]$^+$ (calculated 706.1) with the calculated isotope pattern for that fragment (left). A comparison of **1-Cu**, ^{57}Fe-labelled **1-Cu** and ^{18}O-labelled **1-Cu** (right).*

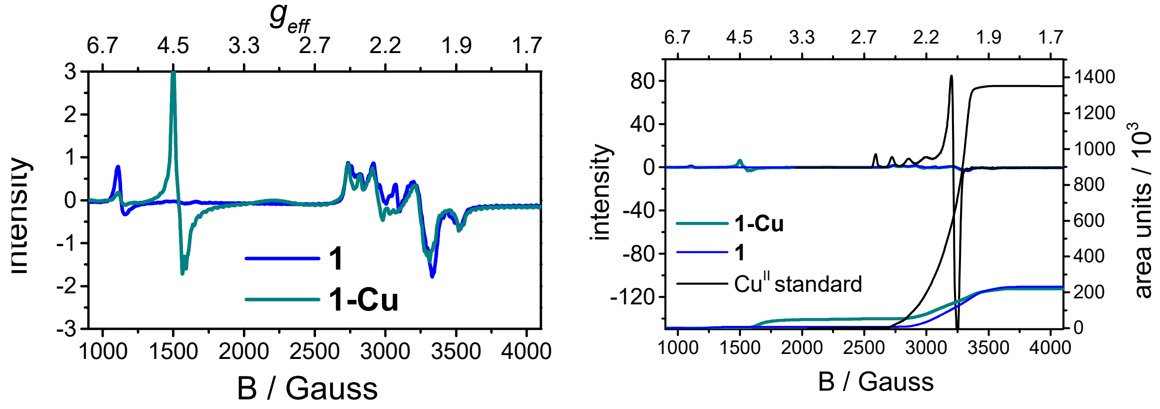

*Figure S 34 X-band EPR spectrum of 1 mM solutions of **1-Cu** and **1** in acetone/CH_3CN 10/1 v/v (left) and comparison to a 1.2 mM Cu^{II} standard ($CuSO_4$ in MeOH) measured under the same conditions for comparison (right) (microwave frequency 9.35 GHz, microwave power 2 mW, modulation amplitude 4.966 G, temperature 12 K) (area units after double integration: 231 000 for **1-Cu** and 221 000 for **1** and 1 360 000 for the Cu^{II} standard)*

3.2. Supplementary Tables S1-S8

Table S 1 DFT models: optimized structures, energies, selected geometric measures, calculated magnetic and vibrational properties, and UV-vis absorption spectra. Relative energies are compared between structures with the same number of atoms.

name	**1-*trans*-b**	**1-*trans*-b-BS** [a]	**1-*trans*-p**	**1-*cis*-b**	**1-*cis*-p**	**2-*trans*-b**	**2-*trans*-p**	**2-*cis*-b**	**2-*cis*-p**
Cu ligand	AN	AN	AN	AN	AN	MeAN	MeAN	MeAN	MeAN
cyclam conf.	*trans*	*trans*	*trans*	*cis*	*cis*	*trans*	*trans*	*cis*	*cis*
AN/MeAN conf.	bent	bent	planar	bent	planar	bent	planar	bent	planar
model	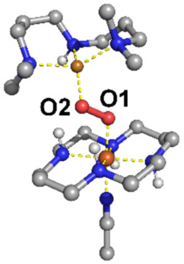	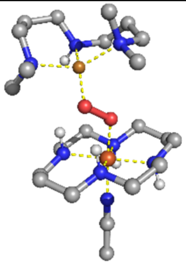	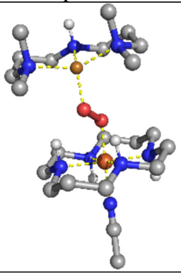	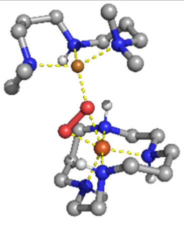	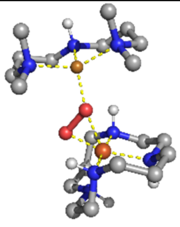	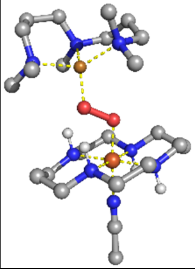	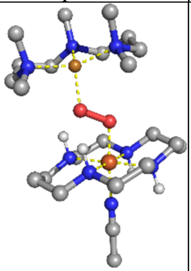	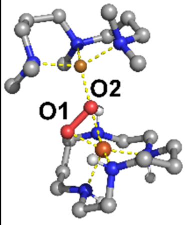	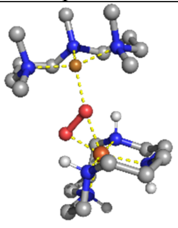
Δ*E* / kcal/mol	0	0.09	0.17	0.43	0	0	0.20	0	0.64
J / cm^{-1}	+46	-74	+106	+157	+133	+109	+116	+185	+97
<u>distances / Å</u>									
Fe-Cu	4.21	4.14	4.27	3.67	3.76	4.30	4.44	3.71	3.94
Fe-O1	1.78	1.79	1.79	1.78	1.78	1.79	1.80	1.78	1.77
Fe-O2	2.72	2.73	2.71	2.01	2.00	2.70	2.69	1.99	1.99
Cu-O1	2.61	2.59	2.56	2.86	2.85	2.72	2.82	2.93	2.95
Cu-O2	1.91	1.92	1.92	2.00	1.99	1.90	1.94	2.00	2.04
O1-O2	1.45	1.44	1.45	1.43	1.44	1.44	1.43	1.43	1.44
<u>angles / °</u>									
Fe-O1-O2	114	116	113	76	76	113	112	76	76
Cu-O2-O1	101	100	98	112	111	108	112	117	115
Fe-O1-O2-Cu	145	137	165	128	138	149	160	130	153
$\nu_{O\text{-}O}(\Delta^{16/18O}\nu_{O\text{-}O})$ / cm^{-1}	737(−37)	735(−39)	738(−38)	875(−43)	852(−48)	750(−40)	767(−40)	862(−49)	853(−48)
O-O Raman int. / a.u.	286	4229	302	n.d.	n.d.	n.d.	n.d.	86	183
$\nu_{Fe\text{-}O}(\Delta^{16/8O}\nu_{Fe\text{-}O})$ / cm^{-1}	618(−29)	627(−28)	608(−28)	565(−18)	578(−19)	619(−30)	616(-−29)	565(−16)	592(−22)
Fe-O Raman int. / a.u.	4398	226	3690	n.d.	n.d.	n.d.	n.d.	38	167
UV-vis absorption	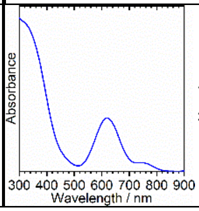	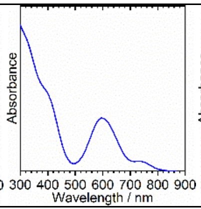	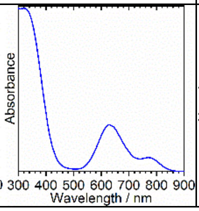	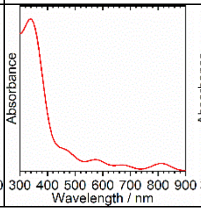	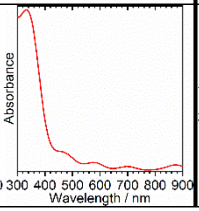	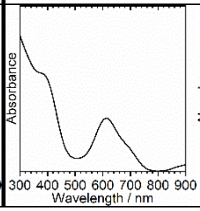	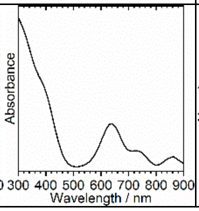	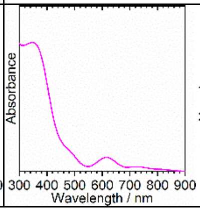	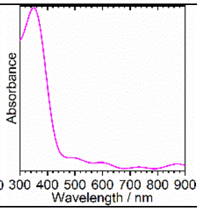

[a] Geometry optimization performed on the BS hypersurface.

Table S 2 Comparison of selected bond lengths derived from EXAFS and calculated by DFT

compound	bond	bond length derived from EXAFS / Å	bond length calculated by DFT / Å
**1** (**1-*trans*-b**)	Fe–O1	1.69 (1.79)[a]	1.78
	Fe–O2	2.47 (2.68)[b]	2.72
	Fe–N_{mean}	1.99	1.99
	Cu–O1	2.57	2.62
	Cu–O2	1.88	1.91
	Cu–N_{mean}	2.02	2.08
**2** (**2-*cis*-b**)	Fe–O1	1.73[c]	1.78
	Fe–O2	2.12[c]	1.99
	Fe–N_{mean}	1.98	2.02
	Cu–O1	2.55	2.93
	Cu–O2	1.84	2.00
	Cu–N_{mean}	2.02	2.09
**1-Cu**	Fe–O1	1.62	
	Fe–N_{mean}	1.99	

[a]Shortest Fe-O bond and (in parenthesis) mean distance for the shorter Fe-O bonds and [b]longer Fe-O bond and (in parenthesis) mean distance of longer Fe-O bond and shorter Fe-C/O distance from two EXAFS simulation approaches in Table S3.
[c]Shortest Fe-O distances from two EXAFS simulation approaches in Table S3.

Table S 3 Iron EXAFS simulation parameters (coordination number, N [per Fe]; interatomic distance, R [Å]; Debye-Waller factor, $2\sigma^2$x10^3 [$Å^2$]; fit error sum (1-3 Å of reduced distance), R_F [%]).

	shell	Fe–N	Fe–O	Fe–O	Fe–Cu/Fe	Fe–Cu/Fe	Fe–C/O	Fe–C	R_F
**1**[a]	N	4*	0.2±0.1	1.8±0.3	0.7±0.2	0.4±0.2	5.7±0.5#	5.3#	6.6
	R	1.99±0.02	1.69±0.05	2.47±0.03	3.30±0.02	4.44±0.03	2.88±0.03	3.49±0.03	
	$2\sigma^2$x10^3	6±2	2*	2*	2*	5*	10*	10*	
**1**[b]	N	4*	0.7±0.3	1.9±0.5	0.7±0.1	0.2±0.2	5.8±0.5#	5.2#	7.1
	R	2.00±0.02	2.18±0.02	2.47±0.02	3.32±0.02	4.47±0.02	2.90±0.01	3.51±0.03	
	$2\sigma^2$x10^3	5±1	2*	2*	2*	5*	10*	10*	
**2**[a]	N	4*	0.3±0.1	1.7±0.2	0.4±0.2	0.6±0.2	5.9±0.4#	5.1#	7.0
	R	1.98±0.02	1.73±0.05	2.48±0.03	3.32±0.02	4.41±0.03	2.89±0.03	3.46±0.04	
	$2\sigma^2$x10^3	7±1	2*	2*	2*	5*	10*	10*	
**2**[b]	N	4*	0.4±0.2	1.50±0.2	0.5±0.1	0.5±0.2	6.0±0.5#	5.0#	8.9
	R	1.99±0.01	2.12±0.02	2.47±0.02	3.34±0.02	4.44±0.03	2.90±0.1	3.49±0.03	
	$2\sigma^2$x10^3	6±2	2*	2*	2*	5*	10*	10*	
**1-Cu**	N	4*	0.5	1.6	0.5	0.2	5.2#	5.8#	8.8
	R	1.99	1.62	2.49	3.29	4.01	2.87	3.47	
	$2\sigma^2$x10^3	8	2*	2*	2*	5*	10*	10*	

*, fixed parameters; #, N-values coupled to a sum of 11. Error values represent the full ranges of fit parameters determined from three independent samples each of **1** and **2**. [a,b] denotes two different simulation approaches for **1** and **2** with a shorter or a longer Fe-O bond to unravel possible iron-oxygen interactions in detail. In tendency, **1** shows a smaller number of shorter and a larger number of longer Fe-O bonds compared to **2**, compatible with prevalence of end-on dioxygen species binding in **1** and of side-on dioxygen species binding in **2.**

Table S 4 Copper EXAFS simulation parameters (coordination number, N [per Cu]; interatomic distance, R [Å]; Debye-Waller factor, $2\sigma^2$x10^3 [$Å^2$]; fit error sum (1-3 Å of reduced distance), R_F [%]).

	shell	Cu–N	Cu–O	Cu–O	Cu–Fe/Cu	Cu–Fe/Cu	Cu–C/O	Cu–C	R_F
**1**	N	3*	0.9±0.3	0.7±0.2	0.8±0.2	0.5±0.2	3.5±0.3#	4.5#	8.1
	R	2.02±0.01	1.88±0.02	2.57±0.01	3.28±0.08	4.38±0.13	2.88±0.02	3.47±0.03	
	$2\sigma^2$x10^3	7±2	2*	2*	5*	10*	9±3#	9#	
**2**	N	3*	0.4±0.2	1.2±0.2	0.5±0.3	0.4±0.2	5.1±0.4#	2.9#	4.4
	R	2.02±0.02	1.84±0.03	2.55±0.02	3.28±0.05	4.40±0.06	2.88±0.04	3.47±0.10	
	$2\sigma^2$x10^3	9±3	2*	2*	5*	10*	13±4#	13#	
	shell	Cu–N/O	Cu–P	Cu–Fe/Cu	Cu–Fe/Cu	Cu–C	Cu–C		
**1-Cu**	N	0.7	3.0	0.1	0.2	2.1	1.7	-	9.1
	R	1.99	2.30	3.10	3.94	2.89	3.37	-	
	$2\sigma^2$x10^3	2*	12	5*	10*	5*	5*	-	

*, fixed parameters; #, N-values coupled to a sum of 8. Error values represent the full ranges of fit parameters determined from three independent samples each of **1** and **2**. The larger number of shorter and smaller number of longer Cu-O bonds in **1** vs. **2** supports different dominating (i.e., side-on vs. end-on) Fe-OO-Cu species in **1** or **2**, in agreement with the iron data. The simulation results for **1-Cu** indicate formation of Cu(I)-triphenylphosphan likely with a further MeCN ligand in the sample.

Table S 5 Results of product characterization and quantification by GC-MS.

Reaction	Products	Yield
**1** + PPh_3	$OPPh_3$	90
**2** + PPh_3		58
[Cu(MeAN)]BF_4 + O_2 + PPh_3		7
[Cu(AN)]BF_4 + O_2 + PPh_3		8
**1-**DCHIm + PPh_3		36
**2-**DCHIm + PPh_3		36
[Cu(MeAN)]BF_4 + DCHIm + O_2 + PPh_3		15
[Cu(AN)]BF_4 + DCHIm +O_2 + PPh_3		12
**1** + 2,6-dtbp	Diketone<br>Dimer-ketone<br>Dimer-OH	10<br>10<br>19
**2** + 2,6-dtbp	Diketone<br>Dimer-ketone<br>Dimer-OH	1<br>4<br>6
**3** + 2,6-dtbp	Diketone<br>Dimer-ketone<br>Dimer-OH	0.2<br>1.2<br>2
[Cu(MeAN)]BF_4 + O_2 + 2,6-dtbp	Diketone<br>Dimer-ketone<br>Dimer-OH	0<br>1.5<br>2
[Cu(AN)]BF_4 + O_2 + 2,6-dtbp	Diketone<br>Dimer-ketone<br>Dimer-OH	0<br>5<br>8
**1**-DCHIm + 2,6-dtbp	Diketone<br>Dimer-ketone<br>Dimer-OH	38<br>212<br>65
**2**-DCHIm + 2,6-dtbp	Diketone<br>Dimer-ketone<br>Dimer-OH	18<br>135<br>13
[Cu(MeAN)]BF_4 + DCHIm + O_2 + 2,6-dtbp	Diketone<br>Dimer-ketone<br>Dimer-OH	7<br>15<br>55
[Cu(AN)]BF_4 + DCHIm +O_2 + 2,6-dtbp	Diketone<br>Dimer-ketone<br>Dimer-OH	7<br>8<br>45
**2** + $^{p\text{-OMe}}$PhOH	not detected	
**1** + $^{p\text{-OMe}}$PhOH	5,5′-Dimethoxy[1,1′-biphenyl]-2,2′-diol	~20
[Cu(MeAN)]BF_4 + DCHIm + O_2 + 2,6-dtbp		~1
[Cu(AN)]BF_4 + DCHIm +O_2 + 2,6-dtbp		~3

Table S 6 Mößbauer parameter of the discussed complexes, intermediates and reaction products.

Assignment	Species	δ / mm s^{-1}	$\lvert\Delta E_Q\rvert$ / mm s^{-1}	yield (main species)
Fe^{III} (S_{Fe} = 1/2)	**1**	0.26	2.11	67%
	**2**	0.24	2.51	79%
	**3** [6]	0.27	2.85	86%
	**1-PhO•**	0.25	2.57	65%
	**1**-DCHIm	0.28	1.87	92%
	**2**-DCHIm	0.29	1.81	79%
Fe^{IV} (S_{Fe} = 1)	[Fe^{IV}(O)(*trans*-cyclam)(CH_3CN)]OTf_2 [6]	0.05	2.49	100%
	[Fe^{IV}(O)(*trans*-cyclam)(DCHIm)]OTf_2	0.05	2.00	90%
	**1-Cu** (**1** + PPh_3)	0.07	2.08	86%
	**2-Cu** (**2** + PPh_3)	0.06	1.97	73%
	**1**-DCHIm + Phenol	0.05	2.00	87%
	**2**-DCHIm + Phenol	0.04	1.99	85%

*Table S 7 Cartesian coordinates of **1-trans-b** in Å.*

O	-1.00352402645558	10.75686169925165	5.86070521597997
O	-1.84351985871243	10.60193118834656	7.02824389893647
C	-4.78476244580775	8.11230044169764	5.64442746721426
N	-3.52955758296922	8.79575078664337	5.23657596221243
C	-3.85765620592292	10.19364009011517	4.86249392058384
N	-1.59608811099470	6.87396820552459	6.70439136907817
H	-0.70115033188390	6.95250907491941	7.20412242095028
C	-2.89764659741075	8.12177216805748	4.06590456510690
N	-2.62984722836257	8.60194922300143	8.97855917123079
C	-2.42535047424079	6.69009779659288	4.31562167000800
C	-1.25474802421262	6.55526696456297	5.28691944241387
C	-2.37055023432594	5.80058792257912	7.38501426771031
C	-2.50364517618226	6.05267165337725	8.88452486110357
C	-3.30247375101585	7.29773019060649	9.26967683193879
C	-1.39722831486021	8.76080379256293	9.78668869673700
C	-3.57138717268878	9.70708152336199	9.29324120570733
Cu	-2.29917294469273	8.74784751746515	6.91553997157034
H	-5.50191222973944	8.09487855112782	4.79915432557404
H	-5.23438292913499	8.65954118053177	6.48776404565924
H	-4.58608777900792	7.07898809045001	5.96096733633713
H	-4.57017344567801	10.19905095179774	4.01424027153015
H	-2.93512774901897	10.71643714256543	4.57544767211414
H	-4.30649589434615	10.70958265873291	5.72436699658655
H	-2.04044237637894	8.75055927971101	3.76512233872523
H	-3.62648744850395	8.12957415425757	3.22839183027585
H	-2.08107849721145	6.29750350017571	3.34121073228834
H	-3.26425088706836	6.03324563692529	4.60967318821188
H	-0.84855831245718	5.52566038624570	5.24456171040531
H	-0.44592715932497	7.24564868627567	4.99123728690575
H	-1.86591041373102	4.83035682190417	7.20968266561072
H	-3.36847500459101	5.73981960204542	6.91878312612791
H	-3.02895919947557	5.18348312602100	9.31988568133470
H	-1.50348565154286	6.06327977243703	9.35792380128408
H	-4.26527033091854	7.30577356734851	8.72787474648429
H	-3.53233166567590	7.26947122033268	10.35528473422815
H	-1.61633801004740	8.59039684853737	10.85962451994236
H	-1.02001431009214	9.78655263953530	9.66870374379710
H	-0.61766973528305	8.05586698660151	9.46138897170158
H	-3.83825841596660	9.69343375304309	10.36906933311027
H	-4.48731805573976	9.58994364513004	8.69280868751022
H	-3.09647014167358	10.66979480927323	9.04914308276110
Fe	0.34496287617579	11.91656862907415	6.07942989497753
N	0.68881508604346	11.17194536782839	7.90258823210751
N	-0.92908021567013	13.30758362827718	6.76601212300496
N	-0.05150284041979	12.66767043791619	4.23417128023885
N	1.59711042336378	10.46360375690873	5.41016061722009
C	0.80627765804620	12.09838011110729	9.05315846613821
C	-0.42237141159512	12.99364092652195	9.19387376230241
C	-0.61698825299104	13.98493897514342	8.05025598431944
C	-1.18122170708928	14.28102271848875	5.66702132428783
C	-1.27656293765890	13.50689759841124	4.36595292939370
C	-0.15285349712647	11.70262886103200	3.10988469477209
C	1.09493593627448	10.83247788022670	2.98548767849584
C	1.25706592431295	9.82016742650994	4.11576177722644
C	1.70404827052823	9.46579371501613	6.51111959809104
C	1.82322283394499	10.21623658920236	7.82323647567520
H	0.94428120037415	11.50237277920874	9.97667188223760
H	1.71877417652456	12.70363024862090	8.90929732266805
H	-1.32983316544114	12.36978205110312	9.30939170243810
H	-0.32024879538483	13.56952285904801	10.13088510642958
H	-1.44098666670880	14.68331503876898	8.29253329479595
H	0.29598484249911	14.58685415374840	7.89706484343598
H	-2.10165605509720	14.86302993312328	5.85775217017608
H	-0.32980732947642	14.98435552714528	5.64192970885612
H	-2.14514412311359	12.82679300123844	4.38704601783600
H	-1.38789853603874	14.18344758910698	3.49899543815859
H	-1.04032373415652	11.07328868048749	3.29606536571392
H	-0.32661485707988	12.26378476626439	2.17164420510781
H	1.02564245104302	10.27221274022983	2.03589782144168
H	2.00005546874494	11.46705419931578	2.90417149921217
H	0.31458385588787	9.26746531983864	4.26960376597372
H	2.04512575962413	9.08447874263866	3.86524450919460
H	0.77722042701096	8.86721554287857	6.49995941693370
H	2.56028804361348	8.78594125581910	6.34960079440939
H	1.81363863454307	9.52157693237037	8.68362670841834
H	2.76295072349747	10.79635673372287	7.86136971838175
H	-0.20266329790643	10.64734270879321	7.99736488583986
H	-1.77723188502948	12.73416420020265	6.90689158401926
H	0.73398683724994	13.29639261919113	4.02487560956711
H	2.51493820914548	10.91389027585818	5.30192208420443
N	1.82413574205662	13.09667989816755	6.20991731050503
C	2.73301627356005	13.82075640404026	6.27690032722277
C	3.85419730453110	14.73049531096108	6.38056693025730
H	3.64113598315812	15.50637158621363	7.13733891931424
H	4.04111977817989	15.21502738900221	5.40598323380253
H	4.75863471139691	14.17314964155292	6.68287321820700

*Table S 8 Cartesian coordinates of **2-cis-b** in Å.*

Fe	-2.39942956573402	12.52856312540343	5.30927700153783
C	-1.34874865272525	14.44964933145374	7.27809924735824
N	-1.93350665401525	14.37803027551847	5.90476519419598
C	-0.04507329923923	13.67339205813659	7.41461056364659
N	-0.67072021408524	11.73651096091202	5.93761823188470
C	-0.19971683324259	12.16081181520565	7.28638803479727
N	-1.53087816004892	12.49183890660817	3.45109180968276
C	0.35519710002491	11.89014888438864	4.86913682241451
N	-4.07989115616893	13.35651562002054	4.55355538235286
C	-0.33231369373609	11.60181099813217	3.55406753400373
C	-2.38400068262473	12.06333746167340	2.30120957654386
C	-3.62872283333806	12.92017357103191	2.11455924595869
C	-4.62198752868703	12.83006639360907	3.26907153012297
C	-3.90338967903052	14.83703393348962	4.55020690048431
C	-3.17530834497131	15.20917067637160	5.82163965289201
H	-1.24116058313828	14.77609356214622	5.25507819309561
H	-0.90816954062895	10.73563078724274	5.98478301469431
H	-1.19510269641339	13.44156106545971	3.23884264061159
H	-4.76468386380789	13.12367088295986	5.28429480502245
H	-1.18043793869847	15.51517495322414	7.52341224601217
H	0.70759212296795	14.06151642516043	6.70271804645859
H	-0.94070731524791	11.78879417627349	8.01264990069294
H	1.20411430264304	11.20603392125609	5.04785368567175
H	-0.69496589801748	10.55834581635275	3.53212517307058
H	-1.76536945900627	12.08563753548386	1.38461490166378
H	-3.34411796735199	13.96915721566163	1.90941509620046
H	-4.91159255659990	11.78166579338063	3.44907071913887
H	-3.30331674556607	15.11865557810656	3.66919011656620
H	-3.79000661717423	14.97036229765335	6.70684041182789
H	-2.92381589786994	16.28404458524946	5.85322877700142
H	-2.11106270304757	14.06173565765137	7.97662312284508
H	0.36184474126929	13.87908622080862	8.42088376536154
H	0.76634829492822	11.66393577706951	7.49525130177449
H	0.73435592473166	12.92559608051508	4.88944143228187
H	0.34326928715646	11.75296926661749	2.69360558126640
H	-2.66398047546572	11.01120939456622	2.48282085096311
H	-4.14292016004193	12.56635559326413	1.20297917422757
H	-5.54208527335170	13.39350801145093	3.02299239049630
H	-4.88144296463381	15.34610861106147	4.47750309167915
C	-4.55129441963500	7.06513335109790	5.46907835995143
N	-3.57792542433638	8.08403129835284	4.99195928428298
O	-3.28524447400978	12.03082632172895	6.77542970007831
O	-3.38152885408857	10.89994724166160	5.90230100131291
C	-4.28426145410268	8.98501395444804	4.05143016245967
N	-1.35459518364408	7.88409723597597	7.28612117082951
C	-2.45103205679845	7.43703716314036	4.25782324886483
N	-3.86593914755723	9.36975322045117	8.52329127715862
C	-1.56593433932182	6.53230364349668	5.10883286419821
C	-0.67140861099875	7.26378668480198	6.10353723715571
C	-1.87187697233515	6.81807393282312	8.19902255577482
C	-2.61920800661306	7.32784288461279	9.43164947016347
C	-3.95544430120682	8.02001752875363	9.16612730924870
C	-3.26985060468241	10.35595779533999	9.45811978697782
C	-5.24511904253838	9.82345865798409	8.19218554190880
Cu	-2.90095003807384	9.12299481875595	6.67965985900857
H	-4.91642207580900	6.45730543150118	4.61683024840056
H	-5.40722895336959	7.57377160538883	5.93870664465462
H	-4.08942463212460	6.39725475696399	6.20924595002651
H	-4.67450100878266	8.40769340854445	3.18990239532799
H	-3.58017692331505	9.74283851719023	3.68245321448403
H	-5.11704181301581	9.48608133787988	4.56636926889045
H	-1.84124485265498	8.25243790785373	3.82750576679817
H	-2.87954083211195	6.85751528726824	3.41332798987889
H	-0.88705089231193	6.00739768839145	4.41175828559206
H	-2.15186693432537	5.73077886983064	5.59254099400120
H	0.10465132577139	6.57022364707760	6.48861728108502
H	-0.14875742007436	8.08340366263728	5.57846794788327
H	-1.00971303299385	6.19684816062499	8.51972114130798
H	-2.54090541595129	6.16476906661707	7.61496620601949
H	-2.84562880165753	6.43590602566983	10.04448823785903
H	-1.96598684768519	7.95103354128970	10.06789174846404
H	-4.57635054684575	7.38773401847237	8.50660241409974
H	-4.50756160201823	8.13401564749787	10.12257782841019
H	-3.89944094158595	10.44328606446295	10.36628074530959
H	-3.20831396693847	11.33306879702285	8.95777358528276
H	-2.25949616484084	10.04911375603023	9.76254243364108
H	-5.86648633711401	9.85557622808241	9.10981891070536
H	-5.70474607431393	9.12690506591505	7.47374490892778
H	-5.20277387953829	10.82824779734168	7.74821799841821
C	-0.31986149295764	8.68181828181336	7.99897018418091
H	-0.76127135621857	9.26463017175953	8.81688632205832
H	0.16095466331743	9.36999775618604	7.28882748120443
H	0.45950691939573	8.01252154669001	8.41459587121317

4. References

(1) Prisecaru, I. *WMOSS4 Mössbauer Spectral Analysis Software*; www.wmoss.org, 2009-2016. (accessed 25.03.2022).
(2) Garcia-Serres, R. *easyMoss : A Python / PyQt5 user-friendly application for the simulation of Mössbauer spectra*; Zenodo, 2021. https://doi.org/10.5281/zenodo.5634948 (accessed 13.03.2024).
(3) Schuth, N.; Mebs, S.; Huwald, D.; Wrzolek, P.; Schwalbe, M.; Hemschemeier, A.; Haumann, M. Effective intermediate-spin iron in O2-transporting heme proteins. *Proc Natl Acad Sci U S A* **2017**, *114* (32), 8556-8561. DOI: 10.1073/pnas.1706527114.
(4) Rehr, J. J.; Kas, J. J.; Vila, F. D.; Prange, M. P.; Jorissen, K. Parameter-free calculations of X-ray spectra with FEFF9. *Physical Chemistry Chemical Physics* **2010**, *12* (21), 5503-5513, 10.1039/B926434E. DOI: 10.1039/B926434E.
(5) Liang, H.-C.; Zhang, C. X.; Henson, M. J.; Sommer, R. D.; Hatwell, K. R.; Kaderli, S.; Zuberbühler, A. D.; Rheingold, A. L.; Solomon, E. I.; Karlin, K. D. Contrasting Copper−Dioxygen Chemistry Arising from Alike Tridentate Alkyltriamine Copper(I) Complexes. *Journal of the American Chemical Society* **2002**, *124* (16), 4170-4171. DOI: 10.1021/ja0125265.
(6) Kass, D.; Corona, T.; Warm, K.; Braun-Cula, B.; Kuhlmann, U.; Bill, E.; Mebs, S.; Swart, M.; Dau, H.; Haumann, M.; et al. Stoichiometric Formation of an Oxoiron(IV) Complex by a Soluble Methane Monooxygenase Type Activation of O_2 at an Iron(II)-Cyclam Center. *Journal of the American Chemical Society* **2020**, *142* (13), 5924-5928. DOI: 10.1021/jacs.9b13756.
(7) Neese, F. The ORCA program system. *WIREs Computational Molecular Science* **2011**, *2* (1), 73-78. DOI: 10.1002/wcms.81.
(8) Neese, F.; Wennmohs, F.; Becker, U.; Riplinger, C. The ORCA quantum chemistry program package. *The Journal of Chemical Physics* **2020**, *152* (22). DOI: 10.1063/5.0004608.
(9) Neese, F. Software update: The ORCA program system—Version 5.0. *WIREs Computational Molecular Science* **2022**, *12* (5). DOI: 10.1002/wcms.1606.
(10) Lenthe, E. v.; Baerends, E. J.; Snijders, J. G. Relativistic regular two-component Hamiltonians. *The Journal of Chemical Physics* **1993**, *99* (6), 4597-4610. DOI: 10.1063/1.466059.
(11) van Lenthe, E.; Baerends, E. J.; Snijders, J. G. Relativistic total energy using regular approximations. *The Journal of Chemical Physics* **1994**, *101* (11), 9783-9792. DOI: 10.1063/1.467943.
(12) van Wüllen, C. Molecular density functional calculations in the regular relativistic approximation: Method, application to coinage metal diatomics, hydrides, fluorides and chlorides, and comparison with first-order relativistic calculations. *The Journal of Chemical Physics* **1998**, *109* (2), 392-399. DOI: 10.1063/1.476576.
(13) Pantazis, D. A.; Chen, X.-Y.; Landis, C. R.; Neese, F. All-Electron Scalar Relativistic Basis Sets for Third-Row Transition Metal Atoms. *Journal of Chemical Theory and Computation* **2008**, *4* (6), 908-919. DOI: 10.1021/ct800047t.
(14) Barone, V.; Cossi, M. Quantum Calculation of Molecular Energies and Energy Gradients in Solution by a Conductor Solvent Model. *The Journal of Physical Chemistry A* **1998**, *102* (11), 1995-2001. DOI: 10.1021/jp9716997.
(15) Grimme, S.; Antony, J.; Ehrlich, S.; Krieg, H. A consistent and accurate ab initio parametrization of density functional dispersion correction (DFT-D) for the 94 elements H-Pu. *The Journal of Chemical Physics* **2010**, *132* (15). DOI: 10.1063/1.3382344.
(16) Perdew, J. P. Density-functional approximation for the correlation energy of the inhomogeneous electron gas. *Physical Review B* **1986**, *33* (12), 8822-8824. DOI: 10.1103/PhysRevB.33.8822.
(17) Becke, A. D. Density-functional exchange-energy approximation with correct asymptotic behavior. *Physical Review A* **1988**, *38* (6), 3098-3100. DOI: 10.1103/PhysRevA.38.3098.
(18) Becke, A. D. Density-functional thermochemistry. III. The role of exact exchange. *The Journal of Chemical Physics* **1993**, *98* (7), 5648-5652. DOI: 10.1063/1.464913.

(19) Lee, C.; Yang, W.; Parr, R. G. Development of the Colle-Salvetti correlation-energy formula into a functional of the electron density. *Phys Rev B Condens Matter* **1988**, *37* (2), 785-789. DOI: 10.1103/physrevb.37.785 From NLM.
(20) Neese, F.; Olbrich, G. Efficient use of the resolution of the identity approximation in time-dependent density functional calculations with hybrid density functionals. *Chemical Physics Letters* **2002**, *362* (1-2), 170-178. DOI: 10.1016/s0009-2614(02)01053-9.
(21) Neese, F.; Wennmohs, F.; Hansen, A.; Becker, U. Efficient, approximate and parallel Hartree–Fock and hybrid DFT calculations. A 'chain-of-spheres' algorithm for the Hartree–Fock exchange. *Chemical Physics* **2009**, *356* (1-3), 98-109. DOI: 10.1016/j.chemphys.2008.10.036.
(22) Yamaguchi, K.; Takahara, Y.; Fueno, T. Ab-Initio Molecular Orbital Studies of Structure and Reactivity of Transition Metal-OXO Compounds. **1986**, 155-184. DOI: 10.1007/978-94-009-4746-7_11.
(23) Martin, R. L. Natural transition orbitals. *The Journal of Chemical Physics* **2003**, *118* (11), 4775-4777. DOI: 10.1063/1.1558471.
(24) Schrodinger, LLC. The PyMOL Molecular Graphics System, Version 1.8. 2015.
(25) Pettersen, E. F.; Goddard, T. D.; Huang, C. C.; Couch, G. S.; Greenblatt, D. M.; Meng, E. C.; Ferrin, T. E. UCSF Chimera—A visualization system for exploratory research and analysis. *Journal of Computational Chemistry* **2004**, *25* (13), 1605-1612. DOI: 10.1002/jcc.20084.
(26) DuBois, J. L.; Mukherjee, P.; Stack, T. D. P.; Hedman, B.; Solomon, E. I.; Hodgson, K. O. A Systematic K-edge X-ray Absorption Spectroscopic Study of Cu(III) Sites. *Journal of the American Chemical Society* **2000**, *122* (24), 5775-5787. DOI: 10.1021/ja993134p.
